# Oestrogen modulates stress‐induced vagally‐dependent gastric responses to oxytocin

**DOI:** 10.1113/JP291603

**Published:** 2026-09-11

**Authors:** Ruchi Bhagat, R. Alberto Travagli, Kirsteen N. Browning

**Affiliations:** ^1^ Department of Neuroscience and Experimental Therapeutics Penn State College of Medicine Hershey Pennsylvania USA; ^2^ Neurobiology Research Newport North Carolina USA

**Keywords:** corticotrophin‐releasing factor, gastric functions, oestrogen, oxytocin, stress, vagus

## Abstract

**Abstract:**

The brain–gut axis is central to gastrointestinal regulation and disorders of gut–brain interaction are more prevalent and often more severe in females. The aim of the present study was to test the hypothesis that brain–gut neurocircuitry is influenced by stress and ovarian hormone levels. Gastric emptying in Sprague–Dawley female rats was slower when oestrogen levels were high, but stress had no further effect. Whole‐cell recordings revealed that, unlike previous reports in male rats, the ‘anti‐stress’ peptide, oxytocin (OXT), suppressed GABAergic transmission to dorsal motor nucleus of the vagus (DMV) neurons under non‐stressed conditions regardless of oestrogen level. Application of astressin, an antagonist of the ‘pro‐stress’ neuropeptide, corticotrophin‐releasing factor (CRF), showed that some presynaptic effects of OXT were dependent upon ongoing CRF1 receptor activity in female vagal neurocircuits. *In vivo* recordings showed that DMV microinjection of OXT decreased gastric tone and motility via vagal efferent pathways involving the release of vasoactive intestinal peptide and nitric oxide onto myenteric neurons, with recruitment of distinct efferent pathways dependent on oestrous stage and stress history. These findings reveal that the oestrous stage and stress do not affect the OXT modulation of inhibitory input to DMV neurons but do determine peripheral effector recruitment. The dissonance between central synaptic modulation and peripheral implementation provides a mechanistic framework for female‐specific variability in gastric motor function and susceptibility to stress‐related gastrointestinal dysfunction.

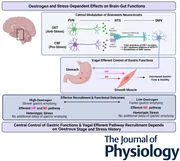

**Key points:**

- Disorders of gut–brain interaction (DGBI) are more prevalent in females. Dorsal motor nucleus of the vagus (DMV) neurons provide extensive extrinsic parasympathetic innervation to the gastrointestinal tract.
- In male rats, the anti‐stress neuropeptide oxytocin (OXT) is known to modulate inhibitory GABAergic transmission to DMV neurons only following stress or activation of pro‐stress corticotrophin‐releasing factor (CRF) receptors.
- The present study demonstrates that, in female rats, OXT suppressed GABAergic transmission to DMV neurons under basal (non‐stressed) conditions in a manner that was independent of oestrous stage, and that CRF receptors were tonically active, even under basal conditions.
- In female rats, parasympathetic control of gastric functions involved non‐adrenergic, non‐cholinergic vagal efferent pathways, with recruitment of distinct pathways dependent on oestrous stage and stress history
- These findings suggest a sex‐specific organization of vagal neurocircuits regulating gastric functions, which may underlie female‐specific variability and susceptibility to stress‐related DGBI.

## Graphical abstract

**Abstract figure legend** In female rats, PVN^CRF^‐DMV neurons and DMV CRF1 receptors are tonically active, allowing oxytocin (OXT) to modulate inhibitory GABAergic transmission to DMV neurons. Parasympathetic control of gastric functions involves engagement of non‐adrenergic non‐cholinergic vagal efferent pathways, but recruitment of distinct pathways depends on oestrous stage and stress history. These findings suggest that central control of gastric functions may underlie female‐specific variability and susceptibility to stress‐related disorders of gut–brain interaction.

## Introduction

The regulation of gastric functions depends on precise integration of intrinsic (enteric) and extrinsic (vagal) innervation to the gastrointestinal (GI) tract, co‐ordinating gastric tone, motility, secretion, and emptying in response to physiological demands, emotional state and environmental stressors. Central to this regulation is the vagus nerve, the primary source of extrinsic parasympathetic innervation to the stomach and the proximal GI tract. Dysregulation of vagal signalling contributes to a range of GI disorders, including functional dyspepsia, gastroparesis and other disorders of gut–brain interaction (DGBI), many of which disproportionately affect women and are strongly associated with symptom exacerbation and psychological stress. Experimental and clinical evidence suggests that ovarian hormones, particularly oestrogen, modulate gastric motility, visceral sensitivity, vagal reflexes and stress responsivity, contributing to delayed gastric emptying and altered autonomic regulation in females (Chen et al., 1995; Jiang, Babic et al., 2019; Meleine & Matricon, 2014).

Within the brainstem, the dorsal vagal complex (DVC), comprising the nucleus tractus solitarius (NTS), dorsal motor nucleus of the vagus (DMV) and area postrema, serves as the principal integrative centre for vagal control of the stomach. Gastric‐projecting DMV neurons regulate smooth muscle tone and motility via postganglionic enteric neurocircuits that utilize both excitatory (acetylcholine) and inhibitory (predominantly nitric oxide (NO) and vasoactive intestinal polypeptide) neurotransmitters, with DMV neuronal activity and vagal efferent output strongly constrained by inhibitory GABAergic inputs from the NTS (Browning et al., 2026). Among the various neuromodulators that act within the DVC, oxytocin (OXT) acts as a critical regulator of autonomic and visceral functions. Beyond its classical role in parturition, lactation and social behaviours, OXT functions as an anti‐stress neuropeptide that modulates gastric function via vagally‐dependent mechanisms (Babygirija et al., 2010; Browning et al., 2026; Neumann, 2002; Raggenbass & Dreifuss, 1992). At the synaptic level, OXT inhibits glutamatergic synaptic transmission in male rats under basal (unstressed) conditions, but inhibits GABAergic transmission only after cAMP levels are elevated; for example, by stress or following exposure to the pro‐stress neuropeptide, corticotrophin‐releasing factor (CRF), providing a direct link between hypothalamic oxytocinergic signalling and peripheral gastric responses (Jiang, Coleman et al., 2018; Peters et al., 2008).

Stress is a major regulator of GI function and a key trigger for symptom exacerbation in DGBI, acting through central stress circuits that engage autonomic and neuroendocrine pathways (Bonaz et al., 2018; Jiang, Greenwood‐Van Meerveld et al., 2019). CRF is involved in mediating these effects at hypothalamic and brainstem sites, including the DVC, where it enhances inhibitory GABAergic input to DMV neurons, suppressing vagal output and gastric motility (Browning et al., 2026; Holmes et al., 2013; Lewis et al., 2002). Chronic stress induces long‐lasting plasticity in autonomic neurocircuits (Carson et al., 2023; Jiang et al., 2022). Chronic homotypic stress (i.e. repeated exposure to the same stress) leads to adaptive plasticity and development of stress resilience, whereas chronic heterotypic stress (i.e. exposure to different stressors) sensitizes the stress response and prevents recovery or adaptation (Bhatnagar & Dallman, 1998; Jiang & Travagli, 2020; Jiang et al., 2022; Jiang, Coleman et al., 2018) and has been linked to persistent GI dysfunction (Browning & Travagli, 2010). Stress‐related GI disorders are more prevalent and severe in women, a vulnerability linked to oestradiol, which modulate gastric motility, vagal reflexes and stress responsiveness, and interacts with OXT and CRF signalling to influence vagal brainstem circuits (Carson et al., 2023; Deussing & Chen, 2018; Hutson et al., 1989; Jiang & Travagli, 2020; McCarthy et al., 1996; Vamvakopoulos & Chrousos, 1993). Most mechanistic studies of oxytocinergic regulation within the DVC have been conducted in males, however, with few specific studies accounting for female‐specific variation in hormonal status.

Despite extensive evidence that OXT, stress, and ovarian hormones each influence GI function, the mechanistic basis by which these signals converge within vagal brainstem circuits to affect gastric functions in females remain poorly defined. In particular, it is unclear whether the OXT‐mediated suppression of inhibitory GABAergic input to gastric‐projecting DMV neurons represents a stable, hormone‐independent synaptic mechanism, or whether it is gated by oestrous stage, circulating oestradiol, or stress history. The aim of the present study was to test the hypothesis that the brain–gut neurocircuitry reveals sex‐specific organization in response to stress and ovarian hormone levels.

## Methods

### Ethical approval

Age‐matched virgin female Sprague‐Dawley rats (*N* = 135; 7–10 weeks of age at the start of experiment) were purchased from Charles River Laboratories (Charles River, Kingston NY, USA) and paired housed in an American Association for the Accreditation of Laboratory Animal Care Accredited Animal Care Facility maintained under a 12:12h light/dark photocycle at 24°C with food and water provided *ad libitum*. All procedures were conducted in accordance with the National Institutes of Health guidelines, following ARRIVE guidelines, and with the approval of the Penn State University College of Medicine Institutional Animal Care and Use Committee (#00420).

### Oestrous cycle determination

In all female rats, the estrous cycle stage was determined on the day of experimentation via vaginal lavage. The oestrogen cycle was categorized as low oestrogen (LE; dioestrus and metoestrus) and high oestrogen (HE; or pro‐oestrus and oestrus) (Gustafsson & Greenwood‐Van Meerveld, 2011), after identification of predominant cell types in the lavage sample. As a result of their relatively lower circulating levels of oestrogen levels, dioestrus and metoestrus were combined as ‘LE’, whereas pro‐oestrus and oestrus were combined as ‘HE’ because of the elevated or peak oestrogen level. This classification provides a functional framework for comparing rats with ovariectomized groups receiving oestrogen replacement via slow‐release capsule containing either a physiologically high or low concentration of 17β‐oestradiol (180 µg mL^−1^ HE or 18 µg mL^−1^ LE, respectively).

### Ovariectomy and 17β‐oestradiol replacement

The female rats were anaesthetized using 2.5% isoflurane mixed with oxygen/CO_2_ (95%/5%) mixture and, once anaesthetized (abolition of the foot pinch withdrawal reflex), the rat was placed on a heating pad to maintain body temperature (37 ± 2°C). Carprofen (Rimadyl; Zoetis, Parsippany, NJ, USA; 5 mg kg^−1^ s.c.) was administered perioperatively, and at 24 h intervals for 3 days following surgery. A 2–3 cm midline incision was made on the dorsal surface to allow access to the abdominal cavity when skin was reflected laterally. The fallopian tubes were identified, sutured and the ovaries removed. The abdominal cavity and skin were sutured in layers (Polymed^@^MT; #100510826, PRN Pharmacal, Pensacola FL) and the rats placed in a warmed cage until recovered from anaesthesia.

### Oestradiol replacement

**Figure A1:**
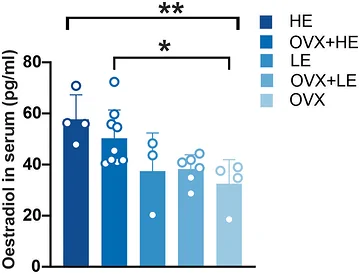
Serum oestradiol level in free cycling and clamped HE rats are higher compared to OVX rats Summary of oestradiol level measured in serum from HE (*N* = 4), OVX + HE (*N* = 9), LE (*N* = 3), OVX + LE (*N* = 6) and OVX (*N* = 4) rats, demonstrating a significant difference in oestradiol levels between groups; specifically, the similarly high level of oestradiol in HE and OVX + HE rats (*N* = number of rats; ^*^ *P* < 0.05, one‐way ANOVA).

Following ovariectomy, a slow‐release capsule containing either a physiologically high or low concentration of 17β‐oestradiol (180 µg mL^−1^, high oestrous or 18 µg mL^−1^, low oestrous, respectively) or vehicle, were placed s.c. within the scapula area (Strom et al., 2012). The ovariectomized rats that received vehicle treatment were designated as OVX, whereas OVX rats receiving high‐or low‐dose oestradiol replacement were designated as OVX + HE and OVX + LE respectively. Serum oestradiol levels were subsequently measured to verify the efficacy of hormone replacement and to confirm classification of groups as free cycling HE, free cycling LE, OVX, OVX + HE and OVX + LE, respectively (see Appendix, Fig. A1).

### Electrophysiology

Female rats were killed following anaesthesia with isoflurane (5% in air) truncal blood was collected via cardiac puncture and processed for plasma collection for later analysis. Following a bilateral pneumothorax, the brainstem was removed and sliced as previously described (Clyburn & Browning, 2021). Briefly, the brainstems were excised quickly and submerged rapidly in cold (4°C) oxygenated Krebs’ solution before being mounted on a vibratome and 300 µm sections throughout the rostro‐caudal extent of the brainstem were cut. Slices were placed in warm (32°C) oxygenated Krebs’ solution for 90 min before recording. For each recording, the brainstem slice was placed in the perfusion chamber on the stage of a trinocular microscope (E600FN; Nikon, Tokyo, Japan). Slices were perfused with warmed Krebs’ solution (in mm: 126 NaCl, 25 NaHCO_3_, 2.5 KCl, 1.2 MgCl_2_, 2.4 CaCl_2_, 1.2 NaH_2_PO_4_ and 10 d‐glucose kept at pH 7.4 by bubbling with 95% O_2_/5% CO_2_) maintained at 32°C at a rate of 2.0–2.5 mL min^−1^. DMV neurons were identified by their size and relative location to the dorsal NTS and ventral hypoglossal nucleus. Electrophysiological recordings were made from DMV neurons, with any one experiment including recordings from a minimum of two or three rats. The recordings were made under bright‐field illumination using 2–4 MΩ patch pipettes filled with a potassium chloride intracellular solution (in mm: 140 KCl, 1 CaCl_2_, 1 MgCl_2_, 10 HEPES, 10 EGTA, 2 NaATP and 0.25 NaGTP adjusted to pH 7.4) and a single electrode voltage‐clamp amplifier (Axopatch 200B; Molecular Devices, San Jose, CA, USA). Data were filtered at 2 kHz, digitized via a Digidata 1440 Interface and stored an analyzed on a PC with pClamp 10 software (Molecular Devices). Recordings with a series resistance larger than 20 MΩ were eliminated from the study.

Miniature inhibitory postsynaptic currents (mIPSCs) were recorded in the voltage‐clamp configuration, at a holding potential of −50 mV, and isolated by perfusion with tetrodotoxin (TTX; 1 µm) and the non‐selective ionotropic glutamate antagonist, kynurenic acid (KynA; 1 mm). All drugs were made freshly in Krebs’ solution on the day of the experiment. Baseline recordings of mIPSCs were made after at least 5 min of TTX + KynA application, followed by application of OXT (Bachem, Bubendorf, Switzerland; H2510‐0025;100 nm) or the CRF1 receptor antagonist, astressin (Bachem; 4026664; 1 µm). Miniature current characteristics, including changes in amplitude (pA) and frequency (p.p.s.), were assessed using the Mini Analysis Program (Synaptosoft, Decatur, GA, USA). A minimum variation of 25% in mIPSC frequency or amplitude was taken as indication of a response.

### Gastric emptying

For gastric emptying (GE) measurement HE (*N* = 10), LE (*N* = 13) and OVX (*N* = 7) rats were selected at random to undergo the ^13^C gastric emptying assay to determine the solid food emptying rate as described previously (Clyburn et al., 2021). Briefly all female rats were first acclimated to the procedure by placing them in their respective testing chamber (VetEquip, Livermore, CA, USA) for ∼3 h per day for 3 consecutive days. During the acclimatization period, rats were fed with 1 g of pancake (De Waffel bakers Buttermilk Pancakes, North Little Rock, AR, USA). After day 3 of acclimatization, rats were fasted overnight, with *ad libitum* access to water, before the testing. On test day, rats were placed in their respective holding chamber for 1 h to allow the carbon dioxide isotope analyzer (Los Gatos Research, San Jose, CA, USA) to equilibrate. Each rat was given 1 g of a pancake that contained 4 µL of [^13^C]‐octanoic acid (Cambridge Isotope Laboratories, Inc., Tewksbury, MA, USA). After visually confirming that rats had eaten the entire pancake (in less than 5 min or they were omitted from the study), the rats were left to remain in their chamber for at least 5 h. During this time, air from each testing chambers was automatically sampled sequentially at 1 s intervals for 30 s every 4 min. At the completion of the gastric emptying study, rats were returned to their cages with full access to food and water. Rats were re‐acclimated to their testing chambers 3–4 days after testing, and gastric emptying was repeated weekly for three baseline measurements.

To analyze GE data, the first 10 s of each 30 s period of data were removed to ensure a complete flush of the previous air sample from the tubing. The remaining 20 s of data were averaged for a single data point for each time interval. The concentration of ^13^CO_2_ was then calculated and expressed as a change over the baseline. The change of concentration of ^13^CO_2_ *vs*. time (*t*) was fitted by a non‐linear regression curve using SigmaPlot (Grafiti LLC, St Palo Alto, CA, USA) with the equation (*y* = *at^b^e* ^−^ *^ct^*), where *y* is the percentage of the ^13^C excretion in the breath per hour (*t*) and *a*, *b* and *c* are regression constants estimated for each breath *vs*. time curve. The gastric half‐emptying time (*T*_1/2_) was calculated from a numerical integration procedure using an inverse gamma function. The three baselines *T*_1/2_ values for each animal were averaged.

### Stress paradigms

The stress procedures were described previously (Jiang & Travagli, 2020; Jiang et al., 2022). Briefly, female rats were randomly divided into three groups: (1) control (no stress); (2) chronic homotypic stress (CHo; in this model rats adapt to stress, i.e. rats become stress resilient); and (3) chronic heterotypic (CHe; in this model rats do not adapt to stress, i.e. rats remain stress‐sensitive). Rats in the control group were handled daily for 10 min without further stress induction. The CHo group underwent 2 h of restraint stress daily, for 5 consecutive days, in a cylinder that did not allow free movement. The CHe group underwent different stressors each day for 5 consecutive days: (1) 2 h restraint; (2) forced swim, i.e. 20 min in a container (length × width × height; 36 × 26 × 24 cm) filled with water at room temperature, such that their hind paws could not touch the bottom of the container; (3) water avoidance, i.e. 90 min on a round platform (6 cm in diameter) in a container with room temperature water at a level just below the top of the platform; (4) cold, i.e. 2 h in a cage maintained at 4°C; and (5) 2 h restraint. The stress treatments were given at the same time period of the day (09.00 h and 11.00 h). Although both day 1 and 5 consisted of restraint stress, the other stressors were applied in a random order. Subsequent experiments were conducted immediately following the final stress treatment on day 5. The number of fecal pellets excreted at day 1 and day 5 during each stress paradigm were noted as a marker of stress response.

### *In vivo* gastric recordings

Gastric tone and motility recordings were made from 37 rats (*N* = 10 control), *N* = 14 CHo and *N* = 13 CHe). Rats were fasted overnight (water *ad libitum*) and anaesthetized with thiobutabarbital (sodium thiobutabartibal hydrate, T33, Inactin; Sigma‐Aldrich, St Louis, MO, USA; 100–150 mg kg^−1^ i.p.). The anaesthesia level (palpebral reflex and/or foot pinch withdrawal reflex) was monitored continuously throughout the experiment, and core temperature was kept at 37°C with a homeothermic heating pad. Once a deep plan of anaesthesia was achieved, rats were intubated with a tracheal catheter, the jugular vein was isolated and a catheter was inserted to allow i.v. injections. A midline laparotomy was performed to expose the anterior gastric wall. Encapsulated miniature strain gauges (6–8 mm; Modern Scientific Research, Roaring Spring, PA, USA) were sutured to the serosal surface of the anterior gastric corpus and antrum in alignment with the gastric circular smooth muscle, and the laparotomy was closed with a 5‐0 suture with the strain gauge leads exteriorized. The signals of the strain gauges were amplified (EXP CLSG‐2; QuantaMetrics, Newton, PA, USA), filtered (low pass cut off = 0.1 Hz; Modern Scientific Research), digitized via a Digidata 1320 interface, and recorded using AxoScope software (Molecular Devices). Rats were then placed in a stereotaxic frame (Kopf Instruments, Tujunga, CA, USA) and the lower medulla was exposed via blunt dissection. The meningeal membrane was removed to expose the vagal trigone. The lower medulla exposed was covered with cotton gauze presoaked in warm saline to stabilize for at least 1 h.

A glass micropipette (20–30 µm tip diameter) was directed into the DVC (in mm: 0.4–0.5 rostro‐caudal from calamus scriptorius, +0.3–0.4 mediolateral from midline, 0.6–0.65 dorsoventral from the brainstem dorsal surface). Drugs were dissolved in phosphate‐buffered saline (PBS) and microinjected in 60 nL volumes over a period of 2 min via a picospritzer (Parker Hannifin, Hollis, NH, USA). Fluorescent microspheres (Fluoresbrite; Polysciences Inc., Warrington, PA, USA) were included in the injectate for *post hoc* verification of the injection site. Gastric tone and motility were monitored for 5 min before drug application and for at least 15 min after the microinjection. Gastric tone and motility were allowed to recover for a minimum of 1 h between injections. Gastric tone was measured as absolute tone variation (in mg) from the baseline. Gastric motility was calculated using a formula described previously (Jiang et al., 2022; Jiang, Browning et al., 2018): motility index = (*N*_1_ × 1 + *N*_2_ × 2 + *N*_3_ × 4 + *N*_4_ × 8)/*t* × 100% where *N* equals the number of peaks in a particular force range (*N*_1_ = 25–50 mg, *N*_2_ = 51–100 mg, *N*_3_ = 101–200 mg, *N*_4_ > 201 mg) and *t* equals the time interval over which the gastric motility was measured.

The effect of drugs on gastric motility was measured relative to the averaged value of gastric motility before microinjection (baseline = 100%). The NO synthase inhibitor nitro‐l‐arginine methyl ester (l‐NAME, 10 mg kg^−1^), the vasoactive intestinal peptide (VIP) antagonist (50 mg kg^−1^) or the muscarinic agonist bethanechol (50 mg kg^−1^) were administered i.v. before the repeated OXT microinjection. At the conclusion of the experiment, rats were killed via administration of a bilateral pneumothorax and transcardial perfusion with 0.1 m PBS followed by paraformaldehyde (PFA, 4%) in 0.1 m PBS. Brainstems were removed and post fixed in 4% PFA for 48 h and then transferred to 0.1 m PBS with 20% sucrose for 2 days. The brainstem was frozen, and coronal sections (50 µm thickness) throughout the rostrocaudal extent of the DVC were cut using a microtome. Every fourth slice was mounted to identify the injection site using a E400 microscope (Nikon).

### Neuronal tracing experiments

Neuronal tracing experiments were carried out in 18 female rats from HE (*N* = 6), LE (*N* = 6) and OVX (*N* = 6) as described previously (Jiang, Coleman et al., 2018). Briefly, rats received an injection of dexamethasone (Sigma, St Aldrich, St Louis, MO, USA; D‐6645; 1 mg kg^−1^ s.c.) prior to being anaesthetized using a mixture of ketamine, xylazine and acepromazine (80 mg kg^−1^, 1.6 mg kg^−1^ and 5 mg kg^−1^, respectively, i.p.), Once a deep plane of anaesthesia was obtained (abolition of the foot pinch withdrawal reflex), rats were placed on a stereotaxic frame (Kopf Instruments, Tujunga, CA, USA) on a homeothermic heating pad to maintain body temperature of 37°C. The dorsal brainstem was exposed via blunt dissection and, following removal of the meningeal membrane, the vagal trigone was exposed. The retrograde tracer, Cholera Toxin B (CTB; List Labs, Campbell, CA, USA; 0.5% in distilled water; 60 nL per injection) was injected into the DMV using a glass pipette attached to a Picospritzer (Toohey Co., Fairfield, NJ, USA) into the rostro‐caudal extent of the DMV with co‐ordinates (in mm) rostro‐caudal 0.0–0.6 mm from calamus scriptorius, medio‐lateral 0.3–0.4 from the midline; dorso‐ventral −0.6 to 0.65 from the surface of the brainstem. Injections were made over 2–3 min and the pipette remain in place for 5 min between injections. The overlying musculature and skin were sutured (5/0 Vicryl suture) and rats returned to their home cages. Carprofen was administered pre‐ and post‐operatively for pain management for 3 days (Rimadyl; Zoetis; 5 mg kg^−1^ s.c.). After 2 weeks of recovery to allow retrograde spread of CTB, rats were anaesthetized (T33, Inactin, dose; 150 mg kg^−1^ i.p.) and, once a deep plane of anaesthesia was reached, were killed via administration of a bilateral pneumothorax and perfused transcardially using 0.9% saline solution followed by 4% PFA in PBS. Fixed brains were dissected and post fixed and cryoprotected for 3 days in a 4% paraformaldehyde + 20% sucrose solution. The hypothalamus and brainstem were sectioned and collected (50 µm thick) throughout their entire rostro‐caudal length using a freezing microtome. Sections were collected in sets of four, and were kept at −20°C in a long‐term storage buffer (50% 0.1 m phosphate buffer (PBS; in mm: 115 NaCl, 75 Na_2_HPO_4_ and 7.5 KH_2_PO_4_): 25% sucrose:25%ethylene glycol) until used for immunofluorescence and immunoenzyme histochemistry.

For immunofluorescence identification, every fourth brainstem slice was used to visualize OXT fibers in the DVC as described previously (Jiang, Coleman et al., 2018). Briefly, free floating slices were washed repeatedly in PBS (0.1 m), followed by incubation in Immunobuffer (0.3% Triton‐X in Tris‐PBS; TPBS) for 1 h. Slices were then incubated in donkey serum (1% in Immunobuffer; Abcam, Cambridge, MA, USA; ab7475) for 1 h to block non‐selective binding before being incubated in goat anti‐choline acetyl transferase [Millipore, Billerica, MA, USA; ab144p (ChAT); dilution 1:1000] and rabbit anti‐OXT (MilliporeSigma, Burlington, MA, USA; ab911; dilution 1:500) primary antibodies for 3 days at room temperature. Following repeated washing in PBS, slices were incubated in donkey anti‐goat AlexaFluor488 and donkey anti‐rabbit AlexaFluor568 (both dilution 1:500; Invitrogen, Carlsbad, CA, USA) secondary antibodies overnight at room temperature. Hypothalamic slices were visualized using the same protocol staining with goat anti‐cholera toxin B [List Labs, Campbell, CA, USA; 703 (CTB): dilution 1:1000] and rabbit anti‐OXT (dilution 1:500) and the same second antibodies. After washing in PBS, brain slices were mounted on subbed slides (Superfrost Microscope Slides; Fisher Scientific, Houston, TX, USA) and cover‐slipped using Fluoromount‐G (Invitrogen).

Immunoenzyme histochemical procedures were as described previously. Every fourth PVN section was rinsed repeatedly with 0.1 m PBS, followed by incubation with immunobuffer (IB) and then IB + 10% normal donkey serum (NDS). Sections were incubated with primary antibody (rabbit anti‐OXT; MilliporeSigma, Burlington, MA, USA: ab911; dilution 1:500) in 1% NDS‐IB for 24 h to 3 days at room temperature on a shaking platform. The sections were then rinsed with TPBS for 3 × 20 min and incubated with 1% NDS‐IB for 1 h. Sections were incubated overnight at room temperature with secondary biotinylated antibodies (donkey anti‐rabbit‐OXT; dilution 1:500). The next day, sections were washed repeatedly with TPBS and incubated with ExtrAvidin horseradish peroxidase (Merck KGaA, Darmstadt, Germany) at a dilution of 1:1500 overnight at room temperature on a shaking platform. The sections were rinsed in TPBS and immunoreactive neurons were visualized with an imidazole‐intensified diaminobenzidine reaction with peroxide generated by glucose oxidase and producing a brown colour reaction. After stopping the reaction with TPBS, the tissues were washed with 0.1 m PBS, before being incubated in IB, and then NDS‐IB before being incubated with second primary antibody (goat anti‐CTB; dilution 1:500) for 3 days. The sections were then rinsed with TPBS before being incubated with biotinylated secondary antibody (donkey anti goat‐CTB) overnight. After washing in TPBS, the slices were finally incubated with ExtrAvidin‐horseradish peroxidase (dilution 1:1500) for 4 h, and colour generated using a Vector SG Substrate kit (Vector Labs, Burlingame, CA, USA) reaction with peroxide generated by glucose oxidase to produce a dark blue reaction. Sections were washed in TPBS, mounted on subbed slides, dehydrated and cover‐slipped with Cytoseal (Thermo Fisher Scientific, Waltham, MA, USA).

### Immunohistochemical measurements

Immunofluorescence staining in PVN and DVC was examined (*N* = 6 in each group) using a Zeiss LSM 900 compact confocal microscope (Zeiss, Oberkochen, Germany). Confocal images throughout the depth of the 50 µm thick slices and images were taken at 20× magnification and compiled to a *z*‐stack. The images were taken from both left and right of the PVN and the DVC. Sequential scanning and adjustment of the appropriate High–Low settings allowed for optimal intensities. All images were taken at the same settings to allow for direct comparison between samples. The intensity of OXT fibers within the DMV or NTS was analyzed using ImageJ software (https://imagej.nih.gov/ij). To determine the intensity of fibers, the DMV or NTS were outlined and background staining was normalized before calculating mean pixel intensity in both the left and right side at the intermediate (Bregma−13.9 mm) level of the brainstem.

The number of OXT and CTB neurons in the PVN were counted using a E600FS microscope (Nikon) and Stereo Investigator software (MBF Bioscience, Williston, VT, USA) using unbiased stereology. Neuronal cell counts were made in both throughout the length of the entire PVN (Bregma levels −0.92 to −2.30 mm). Co‐localization of signal was identified by cells with brown–blue colour and counted as such.

### Oestradiol measurement

Blood was collected from HE (*N* = 4), LE (*N* = 3), OVX (*N* = 4), OVX + HE (*N* = 9) and OVX + LE (*N* = 6) female rats and samples were processed for serum extraction. Rats were killed following anaesthesia with isoflurane (5% in air) and a cardiac puncture was performed to obtain truncal blood samples after which rats received a bilateral pneumothorax. The blood was left to clot for 2–3 h at room temperature before serum was extracted after centrifugation (Eppendorf, Hamburg, Germany; Centrifuge 5417R; 2000 ***g*** for 10 min). Samples were stored at −20°C until use. Serum samples were run in duplicates using the Rat Estradiol Rapid ELISA kit (Thermo Fisher Scientific) at a 1:2 dilution (112.6 pg mL^−1^ sensitivity). The optical density of the samples was measured using a SpectraMax 340PC 384 plate reader (Molecular Devices) interfaced with SoftMaxPRO software (Molecular Devices) at 450 nm. The calibration concentration curve was calculated using an online freely available immunoassay software package utilizing a four‐parameter logistic curve fitting program (https://www.myassays.com) and unknown concentrations were determined via interpolation.

### Statistical analysis

Statistical analysis was conducted using Prism, version 10.0 (GraphPad Software Inc., San Diego, CA, USA) software. Results are expressed as the mean ± SD of the indicated sample size (*N* = rats and *n* = number of neurons). *P* < 0.05 was considered statistically significant.

For neuronal tracing experiments, PVN neurons were identified by immunofluorescence with a recognizable nucleus. Individual OXT and CTB neurons, as well as those containing both OXT and CTB, were counted and total cell counts compared between the groups (HE, LE and OVX) using a one‐way analysis of variance (ANOVA).

Oestradiol levels in serum are reported as pg mL^−1^ based on optical density, and calculated via a concentration curve generated using myassays software. The oestradiol level was measured among five groups (HE, LE, OVX, OVX + HE and OVX + LE) and compared using one‐way ANOVA.

Gastric emptying is expressed as half‐emptying time (min) or as a change from baseline (%), with baseline defined as the average half‐emptying (*T*_1/2_) time of three baseline measurements (taken over the course of 3 weeks). Baseline gastric emptying rates were grouped based on the oestrous stage and compared using one‐way ANOVA.

For electrophysiological results, the magnitude of the response of any neuron to drug application was assessed using one sample paired *t* test against a theoretical baseline of 100%. Paired *t* tests were used to compare responses within neurons before and after the application of drug. The magnitude of response to an individual drug was compared within groups using a two‐tailed unpaired *t* test, whereas the χ^2^ test was used to compare the proportion of responsive neurons between groups. Because analysis was hypothesis driven to answer specific questions rather than for exploratory screening, the correction for multiple comparisons was not applied. A change from baseline greater than 25% was taken to indicate a physiologically significant change. Any neuron that had a baseline mIPSC frequency of less than 0.5 Hz was not included in the analysis.

### Chemicals and drugs

TTX was purchased from Cayman Chemicals (Ann Arbor, Michigan, USA). Unless otherwise stated all the chemicals and drugs were purchased from MilliporeSigma.

## Results

### Properties of mIPSCs in female DMV neurons

The baseline mIPSC frequency of all the neurons recorded from HE, LE, OVX, OVX + HE and OVX + LE female rats were not significantly different from each other (1.9 ± 2.08, 2.3 ± 3.17, 4.0 ± 3.42, 1.9 ± 1.29 and 3.0 ± 2.62 p.p.s., one‐way ANOVA, *F* = 1.43, *P* = 0.228). The baseline mIPSC amplitude of all the neurons recorded from HE, LE, OVX, OVX + HE and OVX + LE female rats were also not significantly different from each other (57.8 ± 16.72, 58.2 ± 18.83, 47.4 ± 17.62, 44.5 ± 12.09 and 45.8 ± 14.22 pA, one‐way ANOVA, *F* = 2.42, *P* = 0.056).

### Oxytocin modulates inhibitory GABAergic neurotransmission to female DMV neurons independent of oestrogen level

**Figure 1:**
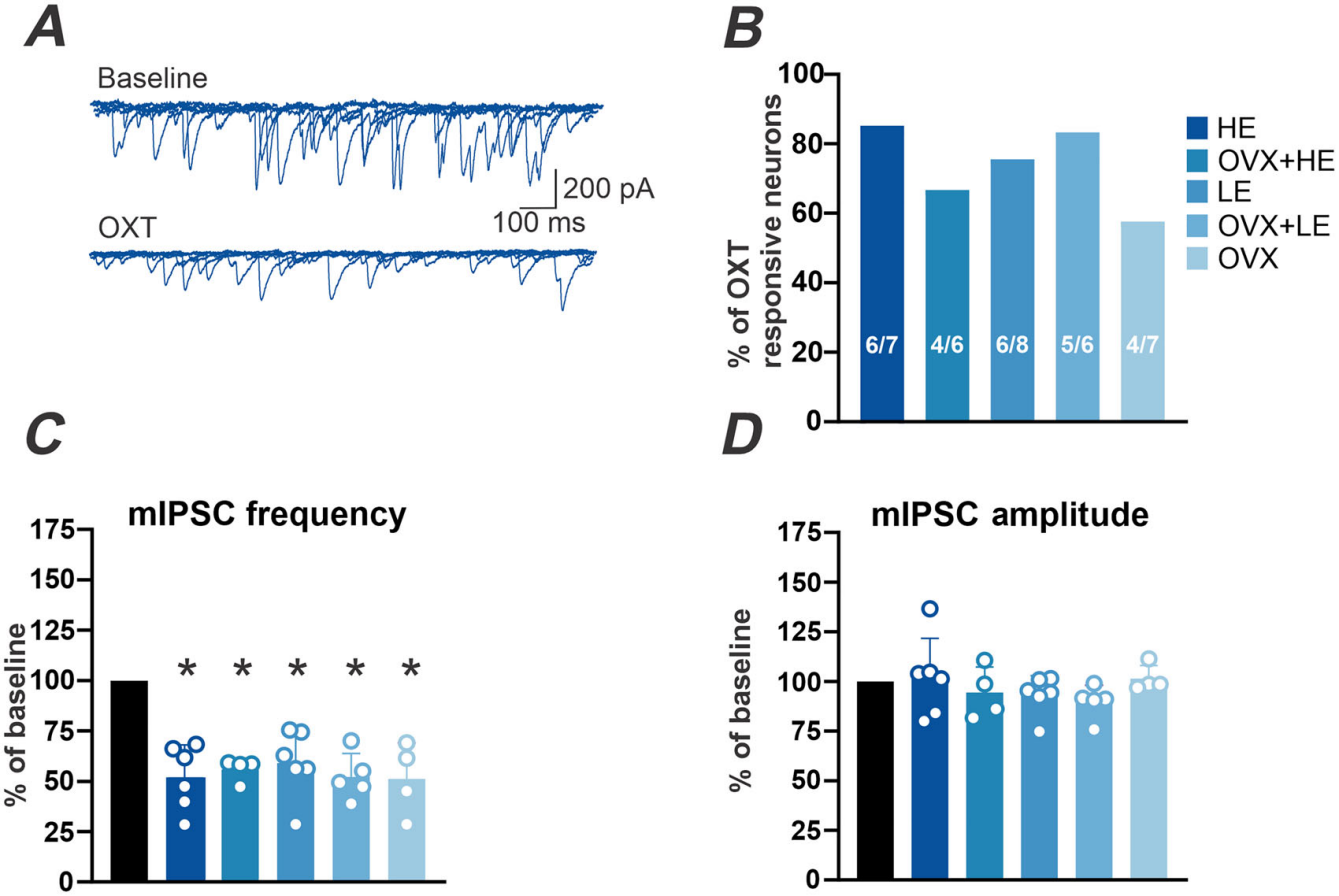
Oxytocin inhibits GABAergic transmission independent of oestrous state *A*, representative overlapping traces of mIPSCs recorded from a HE DMV neuron voltage clamped at –50 mV before (baseline, upper) and after (OXT, lower) superfusion with OXT (100 nm). Note OXT decreased mIPSC frequency. *B*, summary of responsive neurons showing OXT‐induced change in mIPSC frequency at baseline (black) and HE (*n*, *N* = 7, 4), OVX + HE (*n*, *N* = 6, 1), LE (*n*, *N* = 8, 5), OVX + LE (*n*, *N* = 6, 1) and OVX (*n*, *N* = 7, 4) DMV neurons. *C*, summary of mIPSC frequency in response to OXT. *D*, summary of mIPSC amplitude in response to OXT. The results suggest that OXT produces a consistent inhibitory modulation of GABAergic synaptic transmission onto DMV neurons independent of hormonal or oestrous state. *n* = number of neurons and *N* = number of rats; ^*^ *P* < 0.05, paired *t* test compared to baseline.

In six out of seven DMV neurons from HE rats, perfusion of slices with OXT (100 nm) decreased the mIPSC frequency significantly (52 ± 16% of baseline, paired *t* test, *P* = 0.001, *t* = 7.35) (Fig. 1*C* and Table 1) without affecting the amplitude (101.7 ± 20.03% of baseline, paired *t* test, *P* = 0.841, *t* = 0.21) (Fig. 1*D*). In OVX + HE DMV neurons, OXT decreased mIPSC frequency in four out of six DMV neurons (55.8 ± 5.67% of baseline, paired *t* test, *P* = 0.001, *t* = 15.58) without affecting the mIPSC amplitude (94.2 ± 13.04% of baseline, paired *t* test, *P* = 0.442, *t* = 0.88). Comparing HE with OVX + HE DMV neurons, mIPSC frequency (unpaired *t* test, *P* = 0.355, *t* = 0.97) and amplitude (*P* = 0.21, *t* = 1.314) were not different from each other, and the proportion of responsive neurons was unchanged (χ^2^ test, *P* = 0.416).

**Table 1:** Effect of OXT on mIPSC properties in all (responsive) DMV neurons from female rats

Group	*n*, *N* (neurons, rats)	Baseline	+OXT (100 nm)
mIPSC frequency (p.p.s.)			
HE	7, 5	1.1 ± 0.49	0.6 ± 0.23
LE	8, 4	1.7 ± 2.1	1.2 ± 1.21
OVX	7, 3	2.9 ± 2.76	2.1 ± 2.39
OVX + HE	6, 1	1.8 ± 1.35	1.5 ± 0.98
OVX + LE	6, 1	2.9 ± 2.73	1.7 ± 1.44
mIPSC amplitude (pA)			
HE	7, 5	86.1 ± 32.11	91.5 ± 31.04
LE	8, 4	51.3 ± 22.58	45.1 ± 17.43
OVX	7, 3	42.7 ± 7.0	43.5 ± 7.55
OVX + HE	6, 1	42.2 ± 11.32	41.8 ± 8.95
OVX + LE	6, 1	41.6 ± 11.04	37.7 ± 12.61

Similarly, the response of OXT was assessed in LE and OVX + LE rats. In six out of eight neurons from LE rats, perfusion with OXT decreased significantly mIPSC frequency (58.9 ± 17.07% of baseline, paired *t* test, *P* = 0.002, *t* = 5.89) (Table 1) without affecting the amplitude (93.1 ± 9.74% of baseline, paired *t* test, *P* = 0.146, *t* = 1.72). In OVX + LE DMV neurons, five out of six DMV neurons responded to OXT with a decrease in mIPSC frequency, a proportion similar to LE DMV neurons (χ^2^ test, P = 0.91). Furthermore, in OVX + LE, DMV neurons, OXT reduced the mIPSC frequency (52.1 ± 11.61% of baseline, paired *t* test, *P* = 0.001, *t* = 9.21) without affecting the amplitude (89.5 ± 8.47% of baseline, paired *t* test, *P* = 0.045, *t* = 2.76).

The effects of OXT were also assessed in DMV neurons from OVX rats without oestrogen replacement. In four out of seven neurons, OXT decreased mIPSC frequency (51.1 ± 17.95% of baseline, paired *t* test, *P* = 0.012, *t* = 5.45) (Table 1) but not amplitude (101.3 ± 6.72% of baseline, *P* = 0.731, *t* = 0.38).

When comparing the proportion of OXT responsive neurons across all experimental groups (HE, OVX + HE, LE, OVX + LE, OVX), there were no significant differences in proportion of responsive neurons (χ^2^ test *P* = 0.742) or in the magnitude of response to OXT (one‐way ANOVA, *F* = 0.27, *P* = 0.893).

The above results suggest that, in female rats, OXT acted presynaptically to inhibit GABAergic synaptic transmission regardless of oestrogen level or oestrous cycle status.

### Oxytocin modulates inhibitory neurotransmission to female DMV neurons independently of stress status

The baseline mIPSC frequency recorded from HE female that underwent homotypic (CHo) and chronic heterotypic stress (CHe) were not significantly different from each other (1.5 ± 1.56 *vs*. 1.5 ± 1.27 p.p.s., unpaired *t* test, *P* = 0.994, *t* = 0.01).

**Figure 2:**
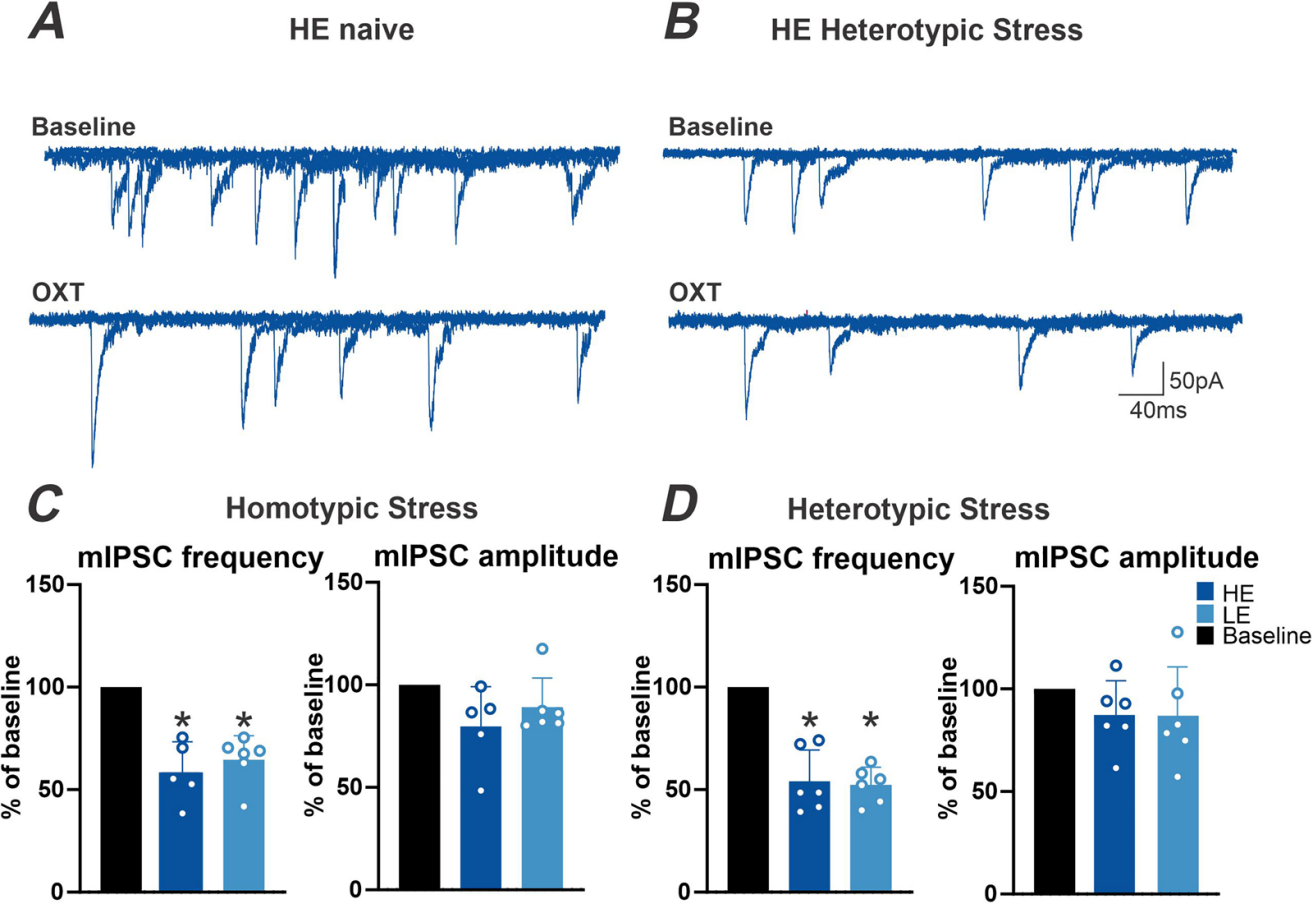
Oxytocin inhibits GABAergic transmission independent of stress *A*, representative overlapping traces of mIPSCs recorded from an unstressed HE DMV neurons voltage clamped at –50 mV before (baseline, upper) and after (lower) OXT application. *B*, representative mIPSCs traces from a HE rats that underwent 5 days of chronic heterotypic stress before (upper) and after (lower) OXT application. *C*, summary of the change in mIPSC frequency and amplitude of DMV neurons from HE (*n*, *N* = 5, 2) and LE (*n*, *N* = 6, 4) in response to OXT following chronic homotypic stress. *D*, summary of the change in mIPSC frequency and amplitude in DMV neurons from HE (*n*, *N* = 6, 3) and LE (*n*, *N* = 6, 4) in response to OXT following chronic heterotypic stress. The results suggest that, in females, NTS‐DMV neurocircuits are sensitive to OXT regardless of oestrogen level or stress. *n* = number of neurons and *N* = number of rats; ^*^ *P* < 0.05, paired *t* test compared to baseline.

In five out of 12 neurons from CHo HE rats, OXT perfusion decreased mIPSC frequency (58.5 ± 14.80% of baseline, paired *t* test, *P* = 0.003, *t* = 6.27) significantly, without affecting amplitude (79.7 ± 19.31% of baseline, paired *t* test, *P* = 0.079, *t* = 2.35) (Fig. 2*C*). In CHe HE, OXT decreased mIPSC frequency in six out of 13 neurons (54 ± 15.27% of baseline, paired *t* test, *P* = 0.007, *t* = 4.38) without affecting the amplitude (87.3 ± 16.61% of baseline, *P* = 0.119, *t* = 0.19) (Fig. 2*D*). The proportion of HE DMV neurons responsive to OXT was not affected by CHo or CHe (χ^2^ test, *P* = 0.823) (Table 2), nor was there a difference in response magnitude between groups (unpaired *t* test, *P* = 0.292, *t* = 1.13*)*.

**Table 2:** Effects of OXT on mIPSC parameters in all (responsive) DMV neurons following chronic homotypic or heterotypic stress

Group	*n*, *N* (neurons, rats)	Baseline	+OXT (100 nm)	*n*, *N* (neurons, rats)	Baseline	+OXT (100 nm)
		Homotypic stress			Heterotypic stress	
mIPSC frequency (p.p.s.)						
HE	5, 2	2.6 ± 1.78	1.4 ± 0.67	6, 3	1.5 ± 1.37	0.9 ± 0.61
LE	6, 4	1.6 ± 1.79	0.9 ± 0.87	6, 4	1.8 ± 1.08	0.9 ± 0.52
mIPSC amplitude (pA)						
HE	5, 2	70.6 ± 14.41	57.7 ± 12.08	6, 3	58.4 ± 10.68	53 ± 12.93
LE	6, 4	74.9 ± 21.21	65.3 ± 14.58	6, 4	69.8 ± 31.80	56.3 ± 18.77

In CHo LE rats, OXT decreased mIPSC frequency in 6 out of 14 neurons (64.5 ± 11.84% of baseline, paired *t* test, *P* = 0.001, *t* = 7.34) without affecting the amplitude (89.1 ± 14.23% of baseline, *P* = 0.118, *t* = 1.89) (Fig. 2*C*,). Similarly, in CHe LE rats, OXT decreased mIPSC frequency in six out of 16 neurons (52.2 ± 8.74% of baseline, paired *t* test, *P* = 0.001, *t* = 13.41) without affecting the amplitude (87 ± 23.77% of baseline, paired *t* test, *P* = 0.814, *t* = 0.27) (Fig. 2*D* and Table 2).

The proportion of LE DMV neurons responsive to OXT was not affected by CHo or CHe (χ^2^ test, *P* = 0.765) (Table 2), but there was a difference in magnitude response between groups (unpaired *t* test, *P* = 0.002, *t* = 4.40).

The results suggest that, unlike male rats (Jiang, Coleman et al., 2018), stress does not modulate the response of female DMV neurons to OXT, regardless of oestrogen level or oestrous cycle stage.

### Astressin modulates the inhibitory GABAergic neurotransmission independent of fluctuations in oestrous level during oestrous cycle

Previous studies (Browning & Travagli, 2010; Browning et al., 2026) have suggested that the ability of OXT to inhibit GABAergic transmission to DMV neurons is modulated by CRF. To determine whether the ability of OXT to decrease GABAergic synaptic transmission is a result of activation of CRF1 receptors, the effects of astressin on mIPSCs was tested.

**Figure 3:**
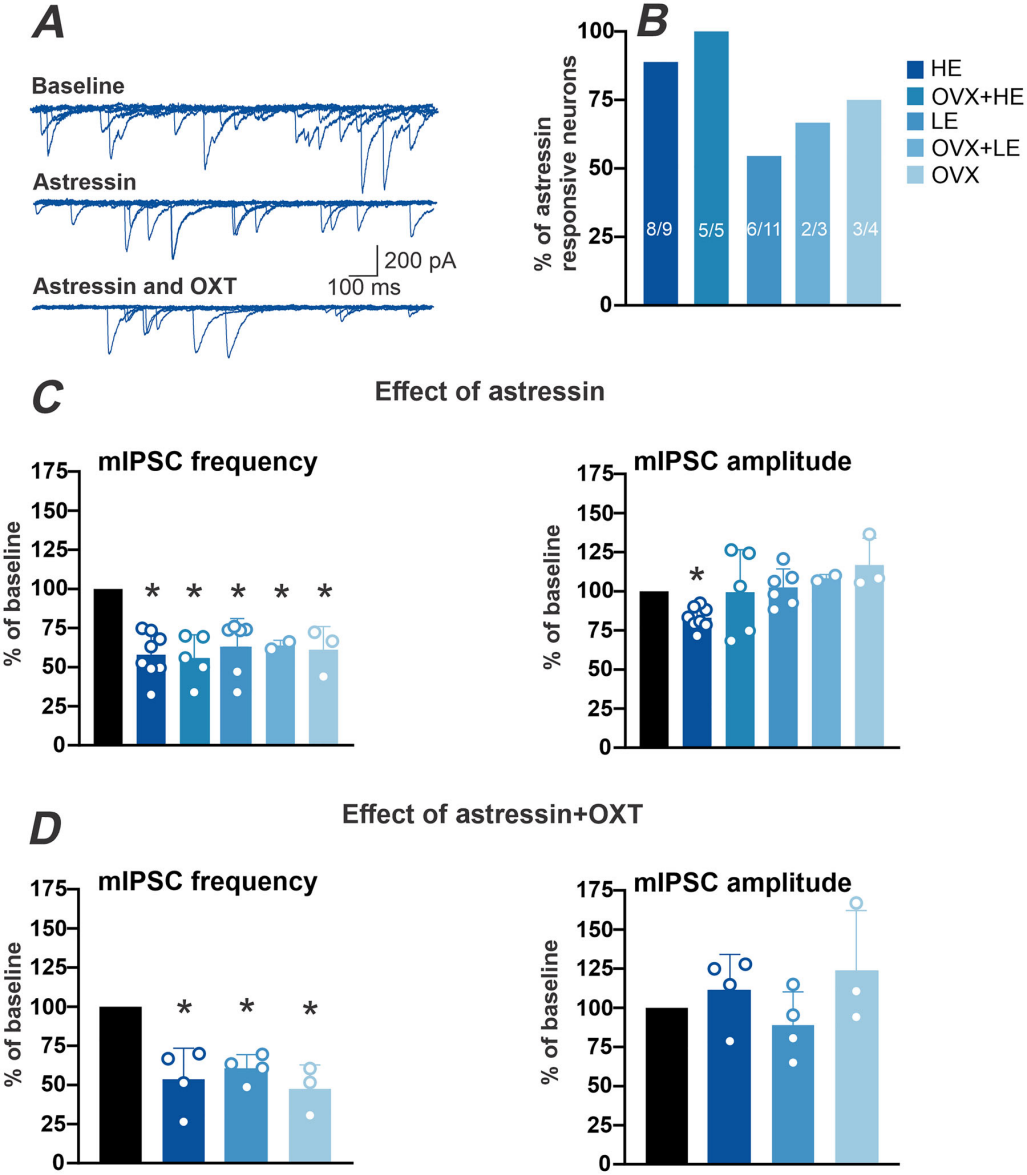
Astressin modulates inhibitory GABAergic neurotransmission independent of oestrogen level of oestrous stage *A*, representative overlapping traces of mIPSCs recorded from an HE DMV neuron voltage clamped at –50 mV at before (baseline upper), in response to astressin application (middle) and following astressin + OXT application (lower). *B*, summary of the proportion of DMV neurons in which astressin modulated mIPSC frequency. *C*, summary of changes in mIPSC frequency (left) and amplitude (right) in DMV neurons in response to astressin application HE (*n*, *N* = 8, 5), OVX + HE (*n*, *N* = 5, 1), LE (*n*, *N* = 6, 5), OVX + LE (*n*, *N* = 2, 1) and OVX alone (*n*, *N* = 3, 2) rats. *D*, summary of changes in mIPSCs frequency and amplitude after astressin + OXT application. The results suggest astressin modulates GABAergic transmission independently of oestrogen level, and the ability of OXT to modulate GABAergic transmission occurs independently of CRF receptor activation. *n* = number of neurons and *N* = number of rats; ^*^ *P* < 0.05, paired *t* test compared to baseline.

In eight out of nine DMV neurons from HE rats, perfusion of slices with astressin (1 µm) decreased the mIPSC frequency (57.7 ± 14.53% of baseline, paired *t* test, *P* = 0.001, *t* = 8.21) and induced a statistically significant, but physiologically non‐significant (i.e. <25% from baseline), change in mIPSC amplitude (83 ± 6.92% of baseline, paired *t* test, *P* = 0.002, *t* = 6.93) (Fig. 3*C* and Table 3). In OVX + HE rats, astressin decreased mIPSC frequency in five out of five DMV neurons (55.7 ± 14.97% of baseline, paired *t* test, *P* = 0.003, *t* = 6.61) without affecting amplitude (99.3 ± 27.10% of baseline, paired *t* test, *P* = 0.652, *t* = 0.51). The proportion of HE and OVX + HE DMV neurons responding to astressin with changes in mIPSC properties did not differ between groups (χ^2^ test, *P* = 0.431), nor did the magnitude of response to astressin (unpaired *t* test, *P* = 0.814, *t* = 0.25).

**Table 3:** Effects of astressin on mIPSC parameters in all (responsive) DMV neurons

Group	*n*, *N* (neurons, rats)	Baseline	+Astressin (1 µm)	*n*, *N* (neurons, rats)	+Astressin (1 µm) and OXT (100 nm)
mIPSC frequency (p.p.s.)					
HE	9, 4	3.9 ± 3.13	2.1 ± 1.84	4, 3	1.1 ± 0.93
LE	11, 5	5.1 ± 5.33	3.8 ± 4.43	4, 3	2.8 ± 2.48
OVX	4, 1	5.6 ± 4.62	4.1 ± 4.35	3, 1	2.8 ± 3.08
OVX + HE	5, 1	2 ± 1.37	1.2 ± 0.90	5, 1	1.3 ± 0.74
OVX + LE	3, 1	3.2 ± 2.96	2.5 ± 2.75	3, 1	1.9 ± 2.44
mIPSC amplitude (pA)					
HE	9, 4	53.9 ± 16.84	44.5 ± 12.89	4, 3	53.9 ± 22.75
LE	11, 5	52.9 ± 17.90	50.1 ± 14.15	4, 3	44.6 ± 22.09
OVX	4, 1	53.1 ± 30.23	58.2 ± 32.20	3, 1	87.7 ± 75.29
OVX + HE	5, 1	47.3 ± 13.69	45.4 ± 13.25	5, 1	42.6 ± 9.03
OVX + LE	3, 1	54.3 ± 18.46	57.2 ± 23	3, 1	78.8 ± 39.7

Similarly, the response to astressin was assessed in LE rats. In LE rats, Astressin significantly decreased mIPSC frequency in six out of 11 neurons (63 ± 17.96% of baseline, paired *t* test, *P* = 0.004, *t* = 5.04) (Fig. 3*C* and Table 3) but not amplitude (102.4 ± 11.9% of baseline, paired *t* test, *P* = 0.787, *t* = 0.29). In OVX + LE rats, astressin reduced the mIPSC frequency in 2/3 neurons (71.4 ± 13.34% of baseline, paired *t* test, *P* = 0.041, *t* = 15.62) without affecting the amplitude (108.3 ± 2.43% of baseline, paired *t* test, *P* = 0.130, *t* = 4.82). The proportion of LE and OVX + LE DMV neurons that responded to astressin with a change in in mIPSC frequency did not differ between groups (χ^2^ test, *P* = 0.707), nor did the magnitude of the response to Astressin (unpaired *t* test, *P* = 0.951, *t* = 0.06).

The above results suggest that astressin acts presynaptically to modulate GABAergic synaptic transmission to DMV neurons in female rats, regardless of oestrogen level of oestrous cycle status. The response to astressin was also assessed in the absence of oestrogen. In OVX rats, astressin decreased mIPSC frequency in three out of four neurons (60.9 ± 14.95% of baseline, paired *t* test, *P* = 0.045, *t* = 4.52) without affecting amplitude (116.7 ± 17.16% of baseline, *P* = 0.233, *t* = 1.69) (Table 3) and unlike male rats (Babic et al., 2011; Babic et al., 2012; Browning & Travagli, 2010; Browning & Travagli, 2011; Browning et al., 2026), CRF1 receptors are tonically active in female rats, even in the absence of external stressors.

Previous studies in male rats have shown that the ability of OXT to act presynaptically to inhibit GABAergic transmission at NTS‐DMV synapses was ‘uncovered’ by stress or CRF exposure (Browning et al., 2026; Carson et al., 2023; Jiang et al., 2022). We therefore assessed whether the action of OXT to modulate GABAergic transmission to female DMV neurons was dependent upon the tonic activation of CRF1 receptors.

In HE rats, in the continued presence of astressin, perfusion with OXT decreased mIPSC frequency in four out of nine neurons (53.5 ± 19.84% of baseline, paired *t* test, *P* = 0.018, *t* = 4.68) without affecting amplitude (111.5 ± 22.63% of baseline, paired *t* test, *P* = 0.384, *t* = 1.02) (Fig. 3*D* and Table 3). When compared to the response of neurons to OXT alone, the magnitude of the inhibition induced by astressin + OXT was unaltered (mIPSC frequency:52 ± 16% *vs*. 53.5 ± 19.84% of baseline; unpaired *t* test, *P* = 0.894, *t* = 0.14, mIPSC amplitude: 101.7 ± 20.03% *vs*. 111.5 ± 22.63% of baseline; unpaired *t* test, *P* = 0.485, *t* = 0.72).

In OVX + HE female rats, however, in the presence of astressin, OXT had no additional effect on mIPSC frequency (127.1 ± 30.32% of baseline) or amplitude (96 ± 10.82% of baseline) (Fig. 3 and Table 3). Thus, the response of OVX + HE DMV neurons to OXT was different to that of astressin + OXT (mIPSC frequency: 55.8 ± 5.67% *vs*. 127.1 ± 30.32% of baseline; unpaired *t* test, *P* = 0.002, *t* = 4.58; mIPSC amplitude: 94.3 ± 13.04% of baseline *vs*. 96 ± 10.82% of baseline; unpaired *t* test, *P* = 0.831, *t* = 0.22).

In LE rats, in the presence of astressin, perfusion with OXT further decreased the mIPSC in four out of 11 neurons (60.5 ± 8.77% of baseline, paired t‐ test, *P* = 0.003, *t* = 8.99) without affecting the amplitude (88.9 ± 21.24% of baseline, paired *t* test, *P* = 0.373, *t* = 1.04) (Fig. 3*D* and Table 3). Thus, the response of DMV neurons in LE rats to OXT were unaffected by astressin (mIPSC frequency: 62.7 ± 22.87% *vs*. 60.5 ± 8.77% of baseline; unpaired *t* test, *P* = 0.860, *t* = 0.18; mIPSC amplitude: 93.2 ± 9.74% *vs*. 88.9 ± 21.24% of baseline; unpaired *t* test, *P* = 0.679, *t* = 0.43).

In OVX + LE female rats, however, OXT had no further effect on mIPSC properties in the presence of astressin frequency (84.8 ± 8.4% of baseline) or amplitude (132.1 ± 23.14% of baseline) (Table 3). Thus, astressin significantly attenuated the ability of OXT to inhibit GABAergic NTS‐DMV transmission in OVX + LE, but not in LE, DMV neurons (mIPSC frequency 84.8 ± 8.4% *vs*. 52.2 ± 11.61% of baseline; unpaired *t* test, *P* = 0.002, *t* = 4.70; mIPSC amplitude 132.1 ± 23.14% *vs*. 89.5 ± 8.47% of baseline; unpaired *t* test, *P* = 0.075, *t* = 3.87).

Finally, in OVX only rats, in the presence of astressin, OXT had effects to further decreased mIPSC frequency (47.5 ± 15.31% of baseline, paired *t* test, *P* = 0.027, *t* = 5.94) without affecting the amplitude (123.9 ± 38.21% of baseline, *P* = 0.391, *t* = 1.08).

Thus, although the proportion of OXT‐responsive DMV neurons was not different in any experimental group (HE, LE, OVX, OVX + HE or OVX + LE (χ^2^; *P* = 0.745), CRF unmasked OXT responses only under conditions of experimentally controlled oestrogen levels following ovariectomy, indicating that oestradiol itself does not directly mediate these effects.

### Oxytocin induces a vagally‐dependent decrease in gastric tone and motility that differs according to stress status

The responses of corpus and antrum tone and motility to brainstem application of OXT were qualitatively similar. Accordingly, the data detailed below are limited to the corpus (Table 4), although the antrum responses are detailed in Table 5.

**Table 4:** OXT‐induced changes in corpus tone and motility in HE and LE rats in naïve, CHo, and CHe stress conditions

HE rats						
Corpus tone (mg)				Corpus motility (% of baseline)		
	Naïve	CHo	CHe	Naive	CHo	CHe
**OXT**	−110.8 ± 62.63 *N* = 10	−67.6 ± 37.5 *N* = 9	−76.8 ± 40.47 *N* = 11	70.9 ± 19.35 *N* = 10	79.9 ± 11.17 *N* = 9	63.9 ± 29.58 *N* = 11
**l‐NAME**	82.3 ± 84.36 *N* = 10	52.6 ± 82.77 *N* = 9	51.8 ± 54.14 *N* = 10	147.6 ± 43.40 *N* = 10	125.1 ± 32.7 *N* = 9	132.2 ± 55.50 *N* = 10
**OXT + l‐NAME**	−86.4 ± 99.22 *N* = 10 *P* = 0.139, *t* = 1.64	−15.2 ± 21.52 *N* = 9 *P* = 0.014, *t* = 3.12	−40.3 ± 34.38 *N* = 10 *P* = 0.012, *t* = 3.16	61.6 ± 19.23 *N* = 10 *P* = 0.193, *t* = 1.42	109.3 ± 31.18^*^ *N* = 9 *P* = 0.032, *t* = 2.56	88.4 ± 26.27^*^ *N* = 10 *P* = 0.047, *t* = 2.50
**VIP antagonist**	50.7 ± 56.47 *N* = 10	31.7 ± 27.81 *N* = 9	36.7 ± 54.51 *N* = 10	116.1 ± 41.15 *N* = 10	106.2 ± 33.89 *N* = 9	119.2 ± 30.75 *N* = 10
**OXT + VIP antagonist**	−46 ± 44.54 *N* = 10 *P* = 0.007, *t* = 3.45	−49.8 ± 49.34 *N* = 9 *P* = 0.350, *t* = 0.99	−32.1 ± 17.77^*^ *N* = 10 *P* = 0.013, *t* = 3.07	90.3 ± 24.13^*^ *N* = 10 *P* = 0.038, *t* = 2.47	90 ± 32.28 *N* = 9 *P* = 0.421, *t* = 0.84	101.4 ± 43.6^*^ *N* = 10 *P* = 0.032, *t* = 2.78
**Bethanechol**	452.2 ± 595.10 *N* = 9	279.2 ± 359.96 *N* = 7	512.4 ± 382.65 *N* = 10	294.4 ± 110.13 *N* = 9	281.9 ± 143.39 *N* = 7	341.6 ± 202.22 *N* = 10
**OXT + bethanechol**	−128.9 ± 96.55 *N* = 9 *P* = 0.659, *t* = 0.41	−79.1 ± 53.69 *N* = 7 *P* = 0.175, *t* = 1.53	−81.3 ± 46.64 *N* = 10 *P* = 0.708, t = 0.39	83.6 ± 26.83 *N* = 9 *P* = 0.214, *t* = 1.35	90.4 ± 23.99 *N* = 7 *P* = 0.459, *t* = 0.82	82.6 ± 22.75 *N* = 10 *P* = 0.459, *t* = 0.82

LE rats						
Corpus tone (mg)				Corpus motility (% of baseline)		
	Naive	CHo	CHe	Naive	CHo	CHe
**OXT**	−84.4 ± 49.35 *N* = 8	−75.3 ± 32.52 *N* = 13	−114.7 ± 55.46 *N* = 10	79.6 ± 10.95 *N* = 8	79.0 ± 22.93 *N* = 13	75.0 ± 12.82 *N* = 10
**l‐NAME**	48.4 ± 55.39 *N* = 8	114.7 ± 134.63 *N* = 12	74.2 ± 49.89 *N* = 10	106.9 ± 17.89 *N* = 8	104.5 ± 29.51 *N* = 12	156.8 ± 54.59 *N* = 10
**OXT + l‐NAME**	−59.2 ± 47.03 *N* = 8 *P* = 0.006, *t* = 3.93	−47.1 ± 37.48 *N* = 12 *P* = 0.036, *t* = 2.38	−73.6 ± 91.07 *N* = 10 *P* = 0.047, *t* = 2.29	93.3 ± 9.26 *N* = 8 *P* = 0.025, *t* = 2.84	108.2 ± 49.56 *N* = 12 *P* = 0.025, *t* = 2.84	73.9 ± 42.85 *N* = 10 *P* = 0.965, *t* = 0.05
**VIP antagonist**	29.0 ± 25.53 *N* = 8	91.6 ± 165.96 *N* = 10	68.7 ± 61.71 *N* = 9	118.5 ± 52.37 *N* = 8	152.3 ± 100.26 *N* = 10	119.6 ± 32.16 *N* = 9
**OXT + VIP antagonist**	−62.8 ± 65.14 *N* = 8 *P* = 0.467, *t* = 0.78	−55.0 ± 52.63 *N* = 10 *P* = 0.101, *t* = 1.83	−76.89 ± 41.40 *N* = 9 *P* = 0.045, *t* = 2.37	81.4 ± 34.83 *N* = 8 *P* = 0.893, *t* = 0.14	81.5 ± 65.95 *N* = 10 *P* = 0.651, *t* = 0.47	86.9 ± 16.07 *N* = 9 *P* = 0.029, *t* = 2.65
**Bethanechol**	244.9 ± 264.21 *N* = 8	832.8 ± 1068.20 *N* = 12	338.4 ± 317.44 *N* = 10	413.0.6 ± 290.48 *N* = 8	502.6 ± 415.12 *N* = 12	412.3 ± 81.06 *N* = 10
**OXT + bethanechol**	−75.9 ± 51.86 *N* = 8 *P* = 0.806, *t* = 0.26	−116.2 ± 140.98 *N* = 12 *P* = 0.306, *t* = 1.07	−155.1 ± 91.78 *N* = 10 *P* = 0.175, *t* = 1.47	92.8 ± 26.10 *N* = 8 *P* = 0.132, *t* = 1.71	93.3 ± 21.94 *N* = 12 *P* = 0.198, *t* = 1.37	65.5 ± 23.62 *N* = 10 *P* = 0.406, *t* = 0.87

*N* = number of rats.

* *P* < 0.05, paired *t* test; OXT *vs*. OXT + l‐NAME, OXT *vs*. OXT + VIP antagonist, OXT *vs*. OXT + bethanechol.

**Table 5:** OXT‐induced changes in antrum tone and motility in HE and LE rats in naïve, CHo and CHe stress conditions

HE						
Antrum tone (mg)				Antrum motility (% of baseline)		
	Naive	CHo	CHe	Naive	CHo	CHe
**OXT**	−135.1 ± 68.52 *N* = 10	−149.8 ± 93.10 *N* = 9	−116.4 ± 75.32 *N* = 10	61.8 ± 22.07 *N* = 10	70.4 ± 10.67 *N* = 9	60.5 ± 28.67 *N* = 11
**l‐NAME**	114.9 ± 107.63 *N* = 10	97.70 ± 96.74 *N* = 9	143.4 ± 119.02 *N* = 10	161.5 ± 72.21 *N* = 10	159.0 ± 92.73 *N* = 9	147.8 ± 69.21 *N* = 10
**OXT + l‐NAME**	−151.4 ± 153.92 *N* = 10 *P* = 0.848, *T* = 0.20	−69.0 ± 57.85 *N* = 9 *P* = 0.282, *t* = 1.18	−62.4 ± 58.62^*^ *N* = 10 *P* = 0.049, *t* = 2.28	66.0 ± 34.56 *N* = 10 *P* = 0.764, *t* = 0.31	107.9 ± 47.00^*^ *N* = 9 *P* = 0.032, *t* = 2.60	111.8 ± 50.63^*^ *N* = 10 *P* = 0.028, *t* = 2.60
**VIP antagonist**	31.3 ± 29.87 *N* = 10	75.6 ± 77.90 *N* = 9	138.8 ± 135.97 *N* = 10	139.9 ± 73.09 *N* = 10	129.5 ± 58.58 *N* = 9	136.7 ± 45.75 *N* = 10
**OXT + VIP antagonist**	−59.4 ± 102.63^*^ *N* = 10 *P* = 0.067, *t* = 2.17	−71.7 ± 115.20^*^ *N* = 9 *P* = 0.035, *t* = 2.70	−87.6 ± 92.79 *N* = 10 *P* = 0.306, *t* = 1.09	104.0 ± 60.75^*^ *N* = 10 *P* = 0.046, *t* = 2.31	108.4 ± 51.86^*^ *N* = 9 *P* = 0.044, *t* = 2.38	74.5 ± 27.70 *N* = 10 *P* = 0.375, *t* = 0.94
**Bethanechol**	513.6 ± 366.14 *N* = 9	586.8 ± 615.91 *N* = 7	963.6 ± 881.29 *N* = 10	528.9 ± 361.90 *N* = 9	533.28 ± 415.59 *N* = 7	430.5 ± 246.84 *N* = 10
**OXT + bethanechol**	−140.6 ± 122.22 *N* = 9 *P* = 0.998, *t* = 0.03	−160.3 ± 112.76 *N* = 7 *P* = 0.153, *t* = 1.64	−246.7 ± 255.25 *N* = 10 *P* = 0.085, *t* = 1.94	92.6 ± 26.16 *N* = 9 *P* = 0.080, *t* = 2.00	82.9 ± 20.73^*^ *N* = 7 *P* = 0.028, *t* = 2.89	85.6 ± 25.06 *N* = 10 *P* = 0.099, *t* = 1.84

LE						
Antrum tone in LE (mg)				Antrum motility in LE (% of baseline)		
	Naive	CHo	CHe	Naive	CHo	CHe
**OXT**	−87 ± 51.14 *N* = 8	−88.7 ± 59.62 *N* = 13	−124.6 ± 39.60 *N* = 11	71.3 ± 18.41 *N* = 8	64.9 ± 24.03 *N* = 13	61.3 ± 31.04 *N* = 11
**l‐NAME**	91.5 ± 93.26 *N* = 8	157.2 ± 125.16 *N* = 12	145.3 ± 159.45 *N* = 10	105.8 ± 37.86 *N* = 8	144.5 ± 71.61 *N* = 12	137.0 ± 47.0 *N* = 10
**OXT + l‐NAME**	−64.4 ± 58.24 *N* = 8 *P* = 0.432, *t* = 0.83	−32.3 ± 48.78^*^ *N* = 12 *P* = 0.011, *t* = 3.07	−72.6 ± 59.34^*^ *N* = 10 *P* = 0.041, *t* = 2.38	107.2 ± 36.03^*^ *N* = 8 *P* = 0.049, *t* = 2.38	93.4 ± 42.48^*^ *N* = 12 *P* = 0.044, *t* = 2.27	75.7 ± 24.71 *N* = 10 *P* = 0.109, *t* = 1.78
**VIP antagonist**	45.3 ± 41.61 *N* = 8	52.6 ± 57.57 *N* = 10	67.9 ± 109.75 *N* = 9	156.0 ± 81.55 *N* = 8	141.1 ± 55.73 *N* = 10	113.2 ± 40.72 *N* = 9
**OXT + VIP antagonist**	−45.8 ± 46.97^*^ *N* = 8 *P* = 0.046, *t* = 2.42	−77.0 ± 74.09 *N* = 10 *P* = 0.880, *t* = 0.15	−83.3 ± 69.15 *N* = 9 *P* = 0.215, *t* = 1.35	91.1 ± 37.51 *N* = 8 *P* = 0.291, *t* = 1.14	73.3 ± 16.88 *N* = 10 *P* = 0.445, *t* = 0.80	88.2 ± 42.87^*^ *N* = 9 *P* = 0.048, *t* = 2.34
**Bethanechol**	602.3 ± 470.56 *N* = 8	730.2 ± 522.84 *N* = 12	825.5 ± 550.77 *N* = 10	647.2 ± 524.26 *N* = 8	474.9 ± 196.69 *N* = 12	828.3 ± 715.10 *N* = 10
**OXT + bethanechol**	−175.3 ± 144.12 *N* = 8 *P* = 0.053, *t* = 2.33	−132.8 ± 97.34 *N* = 12 *P* = 0.140, *t* = 1.59	168.7 ± 116.28 *N* = 10 *P* = 0.108, *t* = 1.79	93.6 ± 23.62 *N* = 8 *P* = 0.080, *t* = 2.04	98.3 ± 35.65^*^ *N* = 12 *P* = 0.005, *t* = 3.52	82.3 ± 31.09 *N* = 10 *P* = 0.056, *t* = 2.19

*N* = number of rats.

* *P* < 0.05, paired *t* test; OXT *vs*. OXT + l‐NAME, OXT *vs*. OXT + VIP antagonist, OXT *vs*. OXT + bethanechol.

In 10 HE rats, microinjection of OXT decreased corpus tone and motility (Table 4). Infusion of the NO synthase antagonist, l‐NAME, itself increased corpus tone and motility; in the presence of l‐NAME, the repeated microinjection of OXT induced an unchanged decrease in corpus tone (paired *t* test, *P* = 0.139, *t* = 1.64) and motility (paired *t* test, *P* = 0.193, *t* = 1.42) (Table 4).

In these same rats, application of the VIP antagonist itself increased corpus tone and motility; in the presence of VIP antagonist, the gastroinhibitory effect of repeated OXT microinjection on corpus tone (paired *t* test, *P* = 0.007, *t* = 3.45) and motility (paired *t* test, P = 0.038, *t* = 2.47) was reduced significantly.

**Figure 4:**
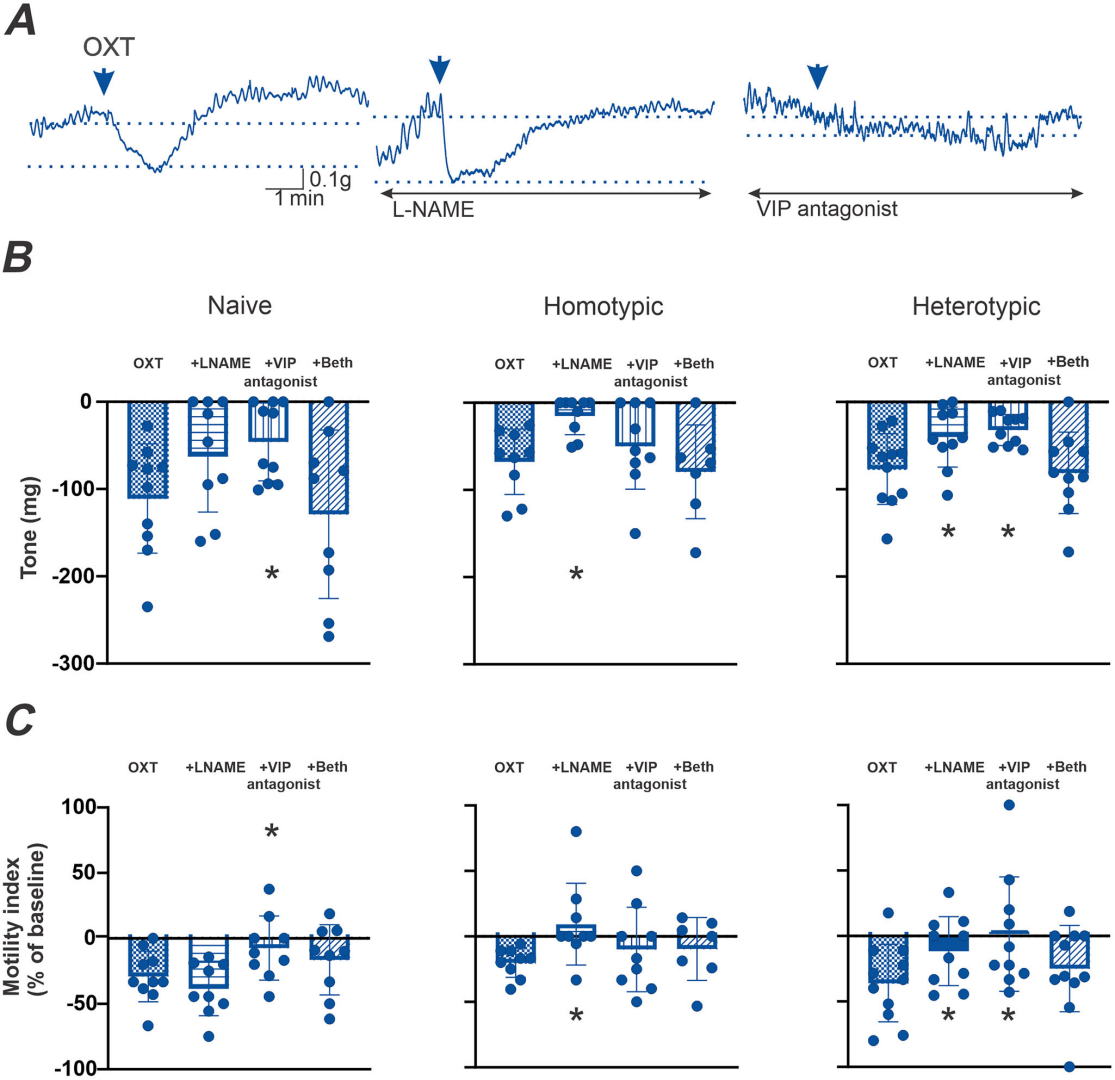
The OXT‐mediated gastroinhibition in HE rats is mediated by postganglionic pathways following stress *A*, representative traces from naïve HE rats showing that microinjection of 150 pmol of OXT in the DMV decreases corpus tone and motility (left trace). Following i.v. administration of nitro‐l‐arginine methyl ester (l‐NAME) the response to repeated OXT was unchanged (middle trace). Intravenous administration of VIP antagonist, however, prevented the gastroinhibition in response to OXT (right trace). *B*, summary of OXT‐induced changes in corpus tone in naïve rats (left) and following chronic homotypic (middle) or heterotypic (right) stress. *C*, summary of OXT‐induced changes in corpus motility in naïve rats (left) and following chronic homotypic (middle) and heterotypic (right) stress. These results suggest that the OXT‐induced gastroinhibition is mediated primarily via a VIP‐dependent postganglionic pathway, but that a NO‐mediated pathway is recruited depending upon oestrous and/or stress. ^*^ *P* < 0.05, paired *t* test compared to OXT alone.

In nine HE rats, infusion with the peripheral muscarinic agonist, bethanechol, increased corpus tone and motility. In the presence of bethanechol (i.e. under conditions where peripheral muscarinic receptors on gastric smooth muscle were maximally activated), the repeated microinjection of OXT induced an unchanged decrease in tone (paired *t* test, *P* = 0.695, *t* = 0.41) and motility (paired *t* test, *P* = 0.214, *t* = 1.35) (Fig. 4 and Table 4)

Following CHo stress, microinjection of OXT in nine HE rats induced a similar decrease in corpus tone and motility (Table 4). Following infusion of the NO synthase inhibitor, l‐NAME, which itself increased corpus tone and motility to a similar degree, the gastroinhibition in response to OXT was significantly attenuated (paired *t* test, *P* = 0.014, *t* = 3.12 for tone and paired *t* test, *P* = 0.032, *t* = 2.56 for motility). In this same group of rats, application of VIP antagonist increased corpus tone but left motility unaffected. In the presence of VIP antagonist, OXT induced a similar gastroinhibition, decreasing corpus tone (paired *t* test, *P* = 0.350, *t* = 0.99) and motility (paired *t* test, *P* = 0.421, *t* = 0.84) (Table 4). In seven of these CHo HE rats, infusion with the peripheral muscarinic agonist, bethanechol, increased corpus tone and motility; in the presence of bethanechol, repeated OXT microinjected induced a similar gastroinhibition (paired *t* test, *P* = 0.175, *t* = 1.53 for tone, and paired *t* test, *P* = 0.459, *t* = 0.82 for motility) (Fig. 4).

In female HE rats that underwent CHe (*N* = 11), microinjection of OXT also decreased corpus tone and motility (Table 4). In 10 of these rats, infusion of l‐NAME, increased corpus tone and motility; in the presence of l‐NAME, the gastroinhibition induced by OXT was attenuated significantly paired *t* test, *P* = 0.012, *t* = 3.16, and motility, paired *t* test, *P* = 0.047, *t* = 2.50). In 10 of these CHe HE rats, application of VIP antagonist increased corpus tone and motility; in the presence of VIP antagonist, the gastroinhibition induced by OXT was significantly attenuated (corpus tone, paired *t* test, *P* = 0.013, *t* = 3.07, and motility, paired *t* test, *P* = 0.032, *t* = 2.78). Finally, in these 10 CHe HE rats, infusion with the peripheral muscarinic agonist, bethanechol, increased corpus tone and motility; in the presence of bethanechol, the gastroinhibition induced by OXT was unaffected (corpus tone, paired *t* test, *P* = 0.708, *t* = 0.39, motility, paired *t* test, *P* = 0.459, *t* = 0.82) (Fig. 4 and Table 4).

These data indicated that, in female rats during periods of high oestrogen levels, the OXT‐induced gastroinhibition occurs via a mechanism that involves the peripheral release of VIP but not NO. Following homotypic stress (CHo), however, the OXT‐mediated gastroinhibition is a result of the peripheral release of NO only, whereas, following heterotypic stress (CHe), the OXT‐mediated gastroinhibition involves peripheral release of both NO and VIP.

In eight LE rats, microinjection of OXT decreased corpus tone and motility (Table 4). Infusion of l‐NAME increased corpus tone but did not affect motility. In the presence of l‐NAME, the gastroinhibition in response to OXT was attenuated (corpus tone, paired *t* test, *P* = 0.006, *t* = 3.93; corpus motility, paired *t* test, *P* = 0.025, *t* = 2.84). In these same rats, application of VIP antagonist increased corpus tone and motility; in the presence of VIP antagonist, the gastroinhibition in response to OXT microinjection was unaffected (corpus tone, paired *t* test, *P* = 0.467, *t* = 0.78; corpus motility, paired *t* test, *P* = 0.893, *t* = 0.14). Infusion of bethanechol increased corpus tone and motility; in the presence of bethanechol, the gastroinhibition in response to OXT microinjection was unaffected (paired *t* test, *P* = 0.806, *t* = 0.26; corpus motility, paired *t* test, *P* = 0.132, *t* = 1.71) (Fig. 4 and Table 4).

Following CHo stress in LE rats, microinjection of OXT (*N* = 13) decreased corpus tone and motility) (Table 4). In 12 of these, infusion of l‐NAME increased corpus tone but not motility; in the presence of l‐NAME, the gastroinhibition induced by OXT was significantly attenuated (paired *t* test, *P* = 0.036, *t* = 2.38; corpus motility, paired *t* test, *P* = 0.025, *t* = 2.83) (Fig. 4 and Table 4). In the same group of rats, application of VIP antagonist increased corpus tone and motility. In the presence of VIP antagonist, the gastroinhibition induced by OXT microinjection was unaffected (corpus tone, paired *t* test, *P* = 0.101, *t* = 1.83, corpus motility, paired *t* test, *P* = 0.651, *t* = 0.47). In these same, infusion with bethanechol increased corpus tone and motility but did not affect the gastroinhibition in response to OXT microinjection (corpus tone, paired *t* test, *P* = 0.306, *t* = 1.107, corpus motility, paired *t* test, *P* = 0.198, *t* = 1.37) (Fig. 4 and Table 4).

Following CHe stress in female LE rats, microinjection of OXT (*N* = 10) decreased corpus tone and motility (Fig. 4 and Table 4). Following infusion of l‐NAME, which increased corpus tone and motility, the gastroinhibition in response to OXT was reduced (corpus tone, paired *t* test, *P* = 0.047, *t* = 2.29; corpus motility, paired *t* test, *P* = 0.965, *t* = 0.05). In nine of these CHe LE rats, the VIP antagonist itself increased corpus tone and motility; in the presence of VIP antagonist, the gastroinhibition induced by OXT was reduced (corpus tone, paired *t* test, *P* = 0.045, *t* = 2.37; corpus motility, paired *t* test, *P* = 0.029, *t* = 2.65). Infusion with the peripheral muscarinic agonist, bethanechol (*N* = 10) increased corpus tone and motility but did not affect the OXT‐induced gastroinhibition (corpus tone, paired *t* test, *P* = 0.175, *t* = 1.47; corpus motility, paired *t* test, *P* = 0.406, *t* = 0.87) (Table 4).

These data suggest that, in LE rats, the OXT mediated gastroinhibition involves peripheral release of NO but, following CHe, the OXT‐dependent gastroinhibition involves peripheral release of VIP instead.

### Oestrogen delays gastric emptying

**Figure 5:**
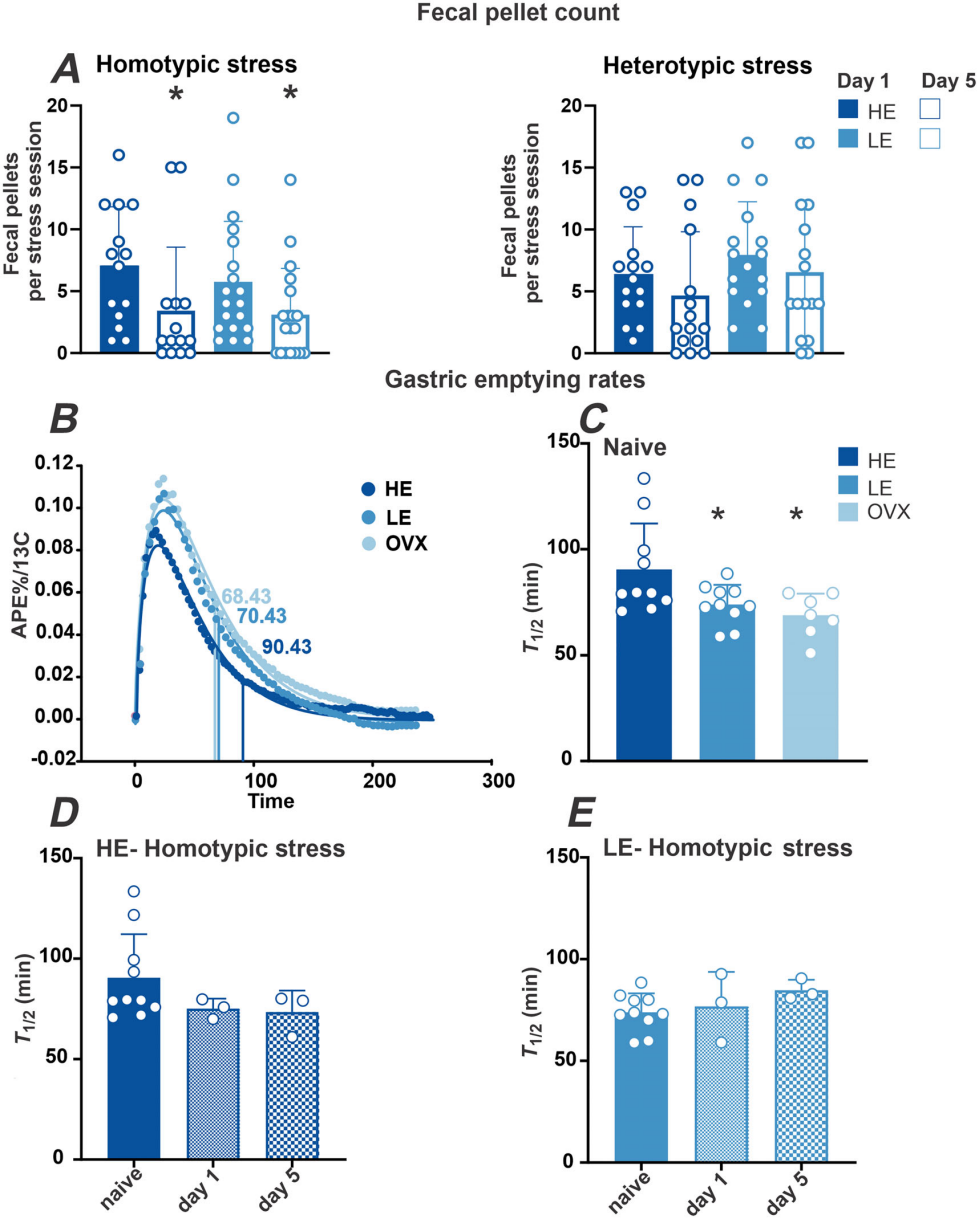
Effects of oestrogen level and stress on fecal pellet count and basal gastric emptying rates *A*, representative traces of fecal pellet count measured at day 1 and day 5 in HE rats after homotypic (left) and heterotypic stress (right), illustrating the adaptation to chronic homotypic, but not heterotypic, stress. ^*^ *P* < 0.05, paired *t* test between day 1 and day 5. *B*, representative gastric emptying curves of HE, LE and OVX rats. Vertical lines indicate the half‐emptying (*T*_1/2_) values. Note the significant delay in gastric emptying rate when oestrogen levels are elevated. *C*, summary of gastric emptying rates in HE (*N* = 10), LE (*N* = 10) and OVX rats (*N* = 7) in naïve female rats. ^*^ *P* < 0.05, one‐way ANOVA comparing *T*_1/2_ values. *D*, summary of gastric emptying rates in HE rats at baseline (stress naïve; *N* = 10) and following acute stress (day 1; *N* = 3) and chronic homotypic stress (*N* = 3). *E*, summary of gastric emptying rates in LE rats at baseline (stress naïve; *N* = 10), and following acute stress (day 1; *N* = 3) and chronic heterotypic stress (day 5; *N* = 3) Data indicate the similar results as observed with HE rats. These results suggest that basal gastric emptying is delayed by HE levels, but that stress exposure has no further effect upon gastric emptying. *N* = number of rats; ^*^ *P* < 0.05, one‐way ANOVA comparing *T*_1/2_ values from day 1 and day 5.

GE rates were assessed in HE (*N* = 10), LE (*N* = 10) and OVX (*N* = 7) rats using the ^13^C octanoic acid breath test. The rate of gastric emptying was similar between OVX (68.7 ± 10.26 min) and LE rats (73.8 ± 9.38 min), but was significantly slower in HE rats (90.4 ± 21.70 min, one‐way ANOVA, *F* = 4.89, *P* = 0.017) (Fig. 5). Acute stress is well recognized to induce a delay in GE in male rats, which is normalized following stress adaptation (CHo) (Jiang et al., 2022). In the present study, however, neither acute nor CHo stress affected GE in HE rats (90.4 ± 21.70 min *vs*. 75.1 ± 4.93 min *vs*. 73.4 ± 10.72 min, respectively; one‐way ANOVA, *F* = 1.41, *P* = 0.279) or in LE rats, (73.8 ± 9.37 *vs*. 76.8 ± 16.88 min *vs*. 84.7 ± 5.09 min, respectively; one‐way ANOVA, *F* = 1.27, *P* = 0.313) (Fig. 5).

These data suggest that, although high oestrogen levels delay GE, female rats do not respond to stress with any change in GE suggesting that, as with the electrophysiology results above, vagal neurocircuits controlling gastric functions in female rats already appear as if in a ‘stressed’ stage.

### Stress increases fecal pellet count in female rats

Fecal pellet count was measured as another indicator of GI responses to stress. In both LE and HE rats, acute stress induced a similar increase in fecal pellet count (6.7 ± 4.23 fecal pellets for HE *vs*. 6.7 ± 4.71 fecal pellets for LE; unpaired *t* test, *P* = 0.987, *t* = 0.02). Following CHo, however, a significant decrease in pellet count was observed from in both HE (day 1, 7.1 ± 4.7 *vs*. day 5, 3.4 ± 5.13 fecal pellets respectively, paired *t* test, *P* = 0.015, *t* = 2.81) and LE rats (day 1, 5.7 ± 4.90 *vs*. day 5, 3.1 ± 3.74 pellets, paired *t* test, *P* = 0.015, *t* = 2.67) (Fig. 5) suggesting that, irrespective of the apparent lack of effect of stress or oestrogen level on gastric outcomes, colonic function showed appropriate responses to acute stress as well as stress adaptation in both LE and HE rats.

By contrast, following CHe stress conditions there was no adaptation in either LE (7.9 ± 4.31 pellets *vs*. 6.5 ± 5.6 pellets for days 1 and 5, respectively, paired *t* test, *P* = 0.312, *t* = 1.05) or HE rats (6.4 ± 3.83 *vs*. 4.6 ± 5.16 pellets for days 1 and 5, respectively, paired *t* test, *P* = 0.257, *t* = 1.18) (Fig. 5). These results suggest that, regardless of oestrogen level or oestrous stage, colonic function (as measured by fecal pellet output) did not show adaptation following heterotypic stress exposure

### Serum oestradiol measurement in female rats

The oestradiol level was measured from blood samples taken from all experimental groups to assess the efficacy and reproducibility of surgical interventions to remove and/or replace oestrogen levels. The HE, LE and OVX rats had serum oestradiol level of 57.7 ± 9.57, 37.4 ± 14.98 and 32.4 ± 9.42 pg mL^−1^, respectively, whereas the serum oestradiol level for clamped OVX + HE and OVX + LE were 50.3 ± 11.04 and 38.2 ± 5.63 pg mL^−1^, respectively. The serum oestradiol level was not significantly different between HE and OVX + HE or between LE and OVX + LE rats. However, a significant overall group effect was observed with significant difference between HE *vs*. OVX as well as HE *vs*. OVX + LE respectively (*P* = 0.006, *F* = 4.87, one‐way ANOVA) (see Appendix, Fig. A1).

The results suggest that artificially clamped oestradiol levels in female rats were comparatively similar to those observed in free cycling rats at high and low oestrus stages.

### Oestrogen level does not alter PVN^OXT^ neuronal count or DVC OXT fibre intensity

**Figure 6:**
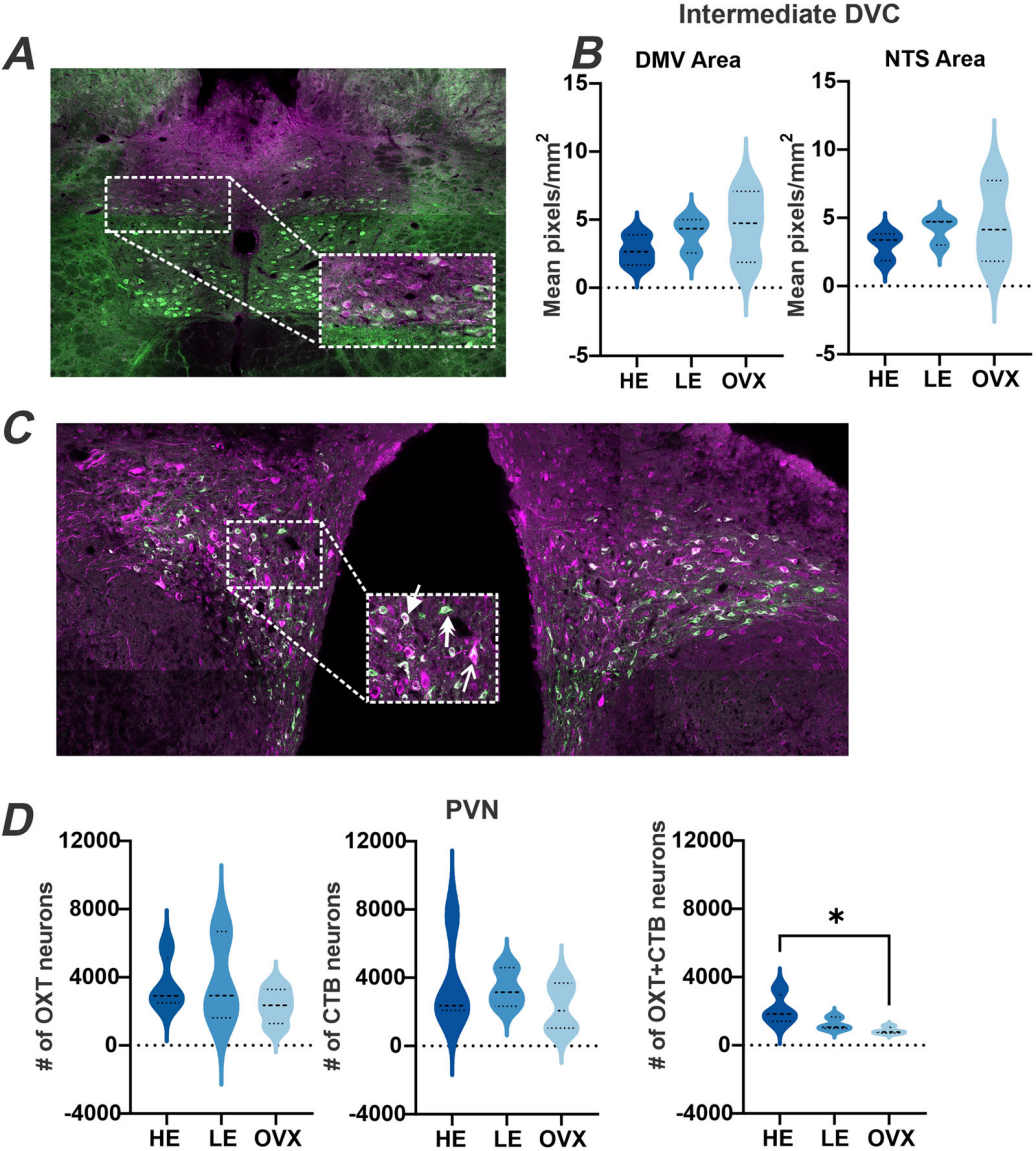
Oestrous stage exerts minor effects on PVN^OXT^‐DMV innervation *A*, representative image of the brainstem containing the DMV [outlined; visualized by ChAT‐immunoreactivity (IR) to identify cholinergic neurons] and NTS in an OVX + HE rats, illustrating OXT fibre density. Magnified insert illustrates ChAT‐IR neurons (green) with adjacent OXT fibers (magenta) Scale bar = 50 µm. *B*, summary of OXT fibre density in the intermediate DMV (left) and NTS (right) illustrating similar innervation density in HE, LE and OVX rats. *C*, representative images of the intermediate PVN in an OVX rat illustrating PVN^CTB^‐DMV neurons (green; double arrow head), PVN^OXT^ neurons (magenta, open arrow head) and PVN^OXT^‐DMV neurons (colocalized OXT and CTB, white, closed arrow head). Magnified insert illustrates PVN^OXT^‐DMV, PVN^CTB^‐DMV and PVN^OXT + CTB^‐DMV neurons). Scale bar = 50 µm. *D*, summary illustrating stereology quantification of PVN^OXT^, PVN^CTB^‐DMV and PVN^OXT^‐DMV neurons showing similar neuronal density in HE, LE and OVX rats. These results suggest that PVN‐OXT to DMV innervation is structurally stable across oestrous stages. ^*^ *P* < 0.05, one‐way ANOVA comparing between the groups.

The PVN provides the only source of OXT innervation to the DVC, and OXT modulates gastric functions, particularly in response to stress (Jiang et al., 2022; Jiang, Coleman et al., 2018; Llewellyn‐Smith et al., 2012) The number of PVN^OXT^ neurons projecting to the DMV was assessed following microinjection of the retrograde tracer, CTB, in HE, LE and OVX rats (*N* = 6 for each group). Using unbiased stereology counting methods, the total number of PVN^OXT^ neurons was similar between HE, LE and OVX rats (3506.0 ± 1543, 3742.0 ± 2628 and 2307.0 ± 1036 cells, respectively, one‐way ANOVA, *F* = 0.72, *P* = 0.513, Figure 6). The number of PVN^CTB^ neurons were also similar between HE, LE and OVX rats (3623 ± 2705, 3356 ± 1146 and 2265 ± 1446 cells, respectively, one‐way ANOVA, *F* = 0.53, *P* = 0.610). The number of co‐localized PVN^OXT/CTB^ neurons was assessed in HE, LE and OVX rats. A significant difference was observed only between HE (2066.0 ± 868.50 cells) and OVX (849.5 ± 188.80 cells) groups (one‐way ANOVA, *F* = 4.65, *P* = 0.046), whereas LE rats showed intermediate values (1237 ± 365.6 cells).

Immunofluorescence assessment of OXT fibre density was measured in both DMV and NTS at the intermediate level (Bregma –13.9 mm) in HE, LE, and OVX rats using ImageJ, and using ChAT‐immunoreactivity to identify cholinergic vagal motoneurons and delineate the boundaries of the DMV. The density of OXT fibers was similar in HE, LE and OVX rats in both the NTS (3 ± 1.03, 4.2 ± 0.98, 4.6 ± 2.98 mean pixels mm^−2^, respectively, one‐way ANOVA, *F* = 0.53, *P* = 0.616) and DMV (2.7 ± 1.11, 3.9 ± 1.27 and 4.6 ± 2.62 mean pixels mm^−2^ respectively, one‐way ANOVA, *F* = 0.80, *P* = 0.491, Figure 6).

Taken together, these results suggest that the number of PVN^OXT^ neurons, as well as OXT innervation of the DMV and NTS, is unaltered by either oestrogen level of oestrous cycle stage, implying that the PVN^OXT^‐DVC innervation is anatomically stable across the oestrous cycle; however, there is modest oestrogen‐dependent change in level of OXT expression in PVN‐DVC neurons

## Discussion

Our data demonstrate that, in female rats, vagal brain–gut neurocircuits are intrinsically configured to a stress‐responsive phenotype, independently of oestrogen level, oestrous cycle stage or external stressors.

The functions of the upper GI tract, including gastric tone and motility, are regulated by the pacemaking activity of vagal preganglionic neurons in the DMV (Travagli et al., 1991). The spontaneous activity of these neurons is strongly shaped by tonic GABAergic inputs arising largely from the NTS (Browning et al., 2026). These inhibitory synapses exhibit pronounced plasticity and are modulated by a variety of conditions, including neuroactive substances, pharmacological manipulation and surgical intervention, as well as both physiological and pathophysiological conditions (Browning et al., 2026). Together, this positions the NTS‐DMV GABAergic neurocircuit as a critical integrative node through which stress‐related neuropeptides and reproductive hormones may regulate parasympathetic control of gastric functions.

Previous work in male rats has shown that OXT does not modulate NTS‐DMV GABAergic transmission under naïve conditions, but that stress, pretreatment with CRF or disruption of vagal afferent input ‘uncovers’ effects of OXT to inhibit GABAergic transmission via mechanisms involving cAMP‐PKA mediated receptor trafficking cAMP (Browning et al., 2026). By contrast, the present study demonstrates that in females (i) OXT suppresses GABAergic transmission at the NTS‐DMV synapse even under basal (unstressed) conditions and (ii) this inhibition is independent of stress exposure, suggesting that (iii) in females, vagal brain–gut circuits are intrinsically configured toward a ‘stressed’ phenotype. Importantly, the presynaptic inhibitory actions of OXT were present regardless of oestrogen level, suggesting that OXT appears to function as a conserved neuromodulator of inhibitory transmission within the DVC regardless of reproductive state. This provides a potential mechanistic explanation as to why circulating ovarian hormone levels alone are poor predictors of DGBI symptom severity in the female population (Sarnoff et al., 2025)

Although the actions of OXT to modulate vagal neurocircuits were remarkably consistent, the physiological consequences of this modulation were highly context dependent. Previous studies demonstrated that, in male rats, OXT induces a gastroinhibition via a vagally‐dependent mechanism involving release of NO (Browning et al., 2013; Browning et al., 2026; Jiang & Travagli, 2020). The present study confirmed the OXT‐mediated gastric relaxation in female rats was also mediated by NO, but only during periods of low oestrogen, whereas VIP pathways were additionally recruited during periods of elevated oestrogen. It should also be noted, however, that oestradiol may modulate gastric motility, tone and emptying via direct actions at the level of the myenteric plexus (Jiang, Greenwood‐Van Meerveld et al., 2019). Oestradiol increases neuronal NO synthase expression and NO release within enteric circuits, thereby enhancing inhibitory NANC signalling.

Furthermore, although stress does not appear to alter the ability of OXT to induce gastroinhibition in females, it may determine the vagal efferent pathway engaged. Such flexibility is consistent with evidence that stress can differentially engage autonomic and neuroendocrine systems, with unpredictable stress producing sustained hypothalamic–pituitary–adrenal axis activation and broader neuromodulatory recruitment (Herman et al., 2003; Ulrich‐Lai & Herman, 2009). This may be especially relevant to the clinical observations of DGBI symptoms such as bloating, altered motility and abdominal pain in female patients which are exacerbated by stress rather than hormonal state alone (Konturek et al., 2011).

In male rats, CRF1 receptors on vagal circuits are inactive under normal conditions but become activated during acute stress. This allows OXT to reduce inhibitory signalling between the NTS and DMV, increasing vagal output and thereby increasing gastric motility and tone (Browning et al., 2026; Jiang et al., 2022). In the present study, however, CRF1 receptors in vagal neurocircuits in female rats were found to be tonically active, even under naïve, unstressed conditions, an effect that was independent of oestrous stage or oestrogen level. Notably, antagonism of CRF1 receptors did not attenuate the presynaptic inhibitory effects of OXT implying that the activation of CRF1 receptors was not responsible for uncovering OXT actions. Following ovariectomy, however, CRF1 antagonism attenuated OXT presynaptic effects, regardless of the oestrogen level. This suggests that sustained oestradiol exposure, regardless of level, may fundamentally alter hypothalamic‐brainstem neuromodulatory integration, consistent with the actions of oestradiol to enhance CRF gene transcription, receptor coupling and downstream signalling under conditions of prolonged stress exposure (Bangasser & Wiersielis, 2018; Bangasser et al., 2010).

Beyond ovarian hormones, however, sex‐linked genetic factors may also contribute to the stability of OXT modulation of vagal circuits. Several components of peptide signalling, including genes involved in synaptic vesicle release, receptor trafficking and G‐protein coupling, are encoded on the X‐chromosome or escape X‐chromosome inactivation, producing sex‐biased expression independently of circulating hormone levels (Arnold & Chen, 2009; Berletch et al., 2011; Raznahan & Disteche, 2021). Such mechanisms may provide a genetic substrate for the persistence of OXT‐dependent effects across oestrous stages and stress conditions (Tukiainen et al., 2017).

In male rats, adaptation to chronic stress and the restoration of appropriate gastric functions, including gastric emptying, involves upregulation of descending PVN^OXT^‐DMV inputs and the CRF‐dependent uncovering of presynaptic OXT effects (Browning & Travagli, 2010; Browning et al., 2026; Jiang et al., 2022). In the present study, however, female rats showed little, if any, adaptation to chronic homotypic stress and gastric emptying rates were unchanged by either acute stress (which would be expected to delay gastric emptying) or chronic homotypic stress (which would be expected to normalize gastric emptying). Rather, our findings suggest that, in females, stress‐related neuroplasticity is expressed primarily at the level of downstream recruitment of vagal efferent effector pathways, rather than through changes in central synaptic sensitivity. This may not only provide a potential mechanistic framework for the persistence and reoccurrence of stress to exacerbate upper GI symptoms in female‐predominant DGBI (Parkman et al., 2011), but also explain the increased dysregulation of gastrointestinal function reported during pregnancy in response to alterations in gonadal hormones, as well as stress and anxiety. Clearly, although potentially translationally relevant, the results of the present study require further validation and testing in rodents, as well as in the clinical population. Notably, however, using fecal pellet output as a surrogate marker of colonic responses to stress suggested that, regardless of oestrogen level, the responses in females are remarkably similar to those observed in male rats. Specifically, acute stress increased fecal pellet output, whereas adaptation to chronic homotypic stress normalized fecal pellet output. This suggests that sex‐dependent differences in extrinsic regulation of the GI tract are more prevalent for gastric *vs*. colonic functions.

### Conclusions

Collectively, these findings support a model in which OXT acts as a stable, oestradiol‐independent modulator of inhibitory synaptic transmission within female vagal brainstem circuits, providing a consistent facilitatory influence on parasympathetic vagal efferent output. Oestrous stage, stress history, CRF signalling and sex‐linked biological factors may also influence how these central signals recruit peripheral gastric motor pathways, promoting the selective engagement of vagal efferent VIP‐ergic, nitrergic, or both, pathways. This multilevel dissociation between central neuromodulation and peripheral implementation may be particularly relevant to the female‐biased prevalence of DGBI, such as functional dyspepsia and irritable bowel syndrome, in which symptoms fluctuate with stress and reproductive state but are not explained fully by circulating ovarian hormone levels alone (Jiang, Greenwood‐Van Meerveld et al., 2019; Mulak et al., 2014; Van Oudenhove et al., 2016). By elucidating how OXT signalling interfaces with stress‐related neuromodulators and oestrous‐dependent effector pathways, the present study provides a mechanistic framework for understanding sex‐specific autonomic regulation and its disruption in GI motility disorders.

## Additional information

## Competing interests

The authors declare that they have no competing interests.

## Author contributions

K.N.B. and A.T. conceived and designed the research. R.B., K.N.B. and A.T. performed experiments, analyzed data, interpreted experimental results, prepared figures, drafted, edited and revised the manuscript, and approved the final version of the manuscript submitted for publication. All authors agree to be accountable for all aspects of the work.

## Funding

This work was supported by NIH grant DK120170 and DK111667.

## Generative AI statement

During the preparation of this manuscript, the authors used ChatGPT to assist in editing. After using this tool/service, the authors reviewed and edited the content as needed and take full responsibility for the content of the published article. All of the data represented in the work were analyzed and quantified without any correction or adaptation via an AI model.

## Supporting information


Peer Review History

## Data Availability

All data are available from the corresponding author upon reasonable request.
