# Supplementary material for: Oestrogen modulates stress‐induced vagally‐dependent gastric responses to oxytocin
Source: J Physiol. 2026 Sep 11;604(18):7570–93. doi: 10.1113/JP291603 (PMC13577686; doi:10.1113/JP291603)
Supplement: Supplementary file 1 [file TJP-604-7570-s001.pdf]

The Journal of Physiology

Estrogen modulates stress-induced vagally-dependent gastric responses to oxytocin

Ruchi Bhagat, R Alberto Travagli, and Kirsteen N Browning
DOI: 10.1113/JP291603

Corresponding author(s): Kirsteen Browning (knb13@psu.edu)

The following individual(s) involved in review of this submission have agreed to reveal their identity: George Nicholas Verne (Referee #1)

Review Timeline:

Submission Date:	20-Apr-2026
Editorial Decision:	20-May-2026
Revision Received:	10-Jun-2026
Editorial Decision:	08-Jul-2026
Revision Received:	24-Jul-2026
Accepted:	12-Aug-2026

Senior Editor: Kim Barrett

Reviewing Editor: Dervla O'Malley

Transaction Report:

(Note: With the exception of the correction of typographical or spelling errors that could be a source of ambiguity, letters and reports are not edited. Depending on transfer agreements, referee reports obtained elsewhere may or may not be included in this compilation. Referee reports are anonymous unless the Referee chooses to sign their reports.)

Dear Dr Browning,

Re: JP-RP-2026-291603 "**Estrogen modulates stress-induced vagally-dependent gastric responses to oxytocin**" by Ruchi Bhagat, R Alberto Travagli, and Kirsteen N Browning

Thank you for submitting your manuscript to The Journal of Physiology. It has been assessed by a Reviewing Editor and by 2 expert referees and we are pleased to tell you that it is acceptable for publication following satisfactory revision.

The referee reports are copied at the end of this email.

Please address all the points raised and incorporate all requested revisions or explain in your Response to Referees why a change has not been made. We hope you will find the comments helpful and that you will be able to return your revised manuscript within 4 weeks. If you require longer than this, please contact journal staff: jp@physoc.org.

Your revised manuscript should be submitted online using the link in your Author Tasks Link Not Available. This link is accessible via your account as Corresponding Author; it is not available to your co-authors. If this presents a problem, please contact journal staff (jp@physoc.org). Image files from the previous version are retained on the system. Please ensure you replace or remove any files that are being revised.

If you do not wish to submit a revised version of your manuscript, you must inform our journal staff (jp@physoc.org) or reply to this email to request withdrawal. Please note that a manuscript must be formally withdrawn from the peer review process at one journal before it may be submitted to another journal.

TRANSPARENT PEER REVIEW POLICY: To improve the transparency of its peer review process The Journal of Physiology publishes online, as supporting information, the peer review history of all articles accepted for publication. Readers will have access to decision letters, including Editors' comments and referee reports, for each version of the manuscript, as well as any author responses to peer review comments. Referees can decide whether or not they wish to be named on the peer review history document.

ABSTRACT FIGURES: Authors are expected to use The Journal's premium BioRender account to create/redraw their Abstract Figures. Information on how to access this account is here: https://physoc.onlinelibrary.wiley.com/journal/14697793/biorender-access.

This will enable Authors to create and download high-resolution figures. If authors have used the free BioRender service, they can use the instructions provided in the link above to download a high-resolution version suitable for publication.

The link provided should only be used for the purposes of this submission. Authors will be charged for figures created on this account if they are not related to this manuscript submission.

LANGUAGE EDITING AND SUPPORT FOR PUBLICATION: If you would like help with English language editing, or other article preparation support, Wiley Editing Services offers expert help, including English Language Editing, as well as translation, manuscript formatting, and figure formatting at www.wileyauthors.com/eeo/preparation. You can also find resources for Preparing Your Article for general guidance about writing and preparing your manuscript at www.wileyauthors.com/eeo/prepresources.

RIGOUR AND REPRODUCIBILITY: We recommend authors consult The Journal's Rigour and Reproducibility requirements to ensure that their manuscript is suitable for publication: https://physoc.onlinelibrary.wiley.com/pb-assets/hub-assets/physoc/documents/TJP-Rigour-and-Reproducibility-Requirements.pdf

REVISION CHECKLIST:

Check that your Methods section conforms to journal policy: https://jp.msubmit.net/cgi-bin/main.plex?form_type=display_requirements#methods

Check that data presented conforms to the statistics policy: https://jp.msubmit.net/cgi-bin/main.plex?form_type=display_requirements#statistics

Upload a full Response to Referees file. To create your 'Response to Referees' copy all the reports, including any comments from the Senior and Reviewing Editors, into a Microsoft Word, or similar, file and respond to each point, using font or background colour to distinguish comments and responses and upload as the required file type.

Please upload two versions of your manuscript text: one with all relevant changes highlighted and one clean version with no changes tracked. The manuscript file should include all tables and figure legends, but each figure/graph should be uploaded as separate, high-resolution files. The journal is now integrated with Wiley's Image Checking service. For further details, see: https://www.wiley.com/en-us/network/publishing/research-publishing/trending-stories/upholding-image-integrity-wileys-image-screening-service

You may also upload:

- 'Potential Cover Art' for consideration as the issue's cover image
- Appropriate Supporting Information (Video, audio or data set: see https://jp.msubmit.net/cgi-bin/main.plex?form_type=display_requirements#supp).

We look forward to receiving your revised submission.

If you have any queries, please reply to this email and we will be pleased to advise.

Yours sincerely,

Kim Barrett
Senior Editor
The Journal of Physiology

REQUIRED ITEMS

- Author photo and profile. First or joint first authors are asked to provide a short biography (no more than 100 words for one author or 150 words in total for joint first authors) and a portrait photograph. These should be uploaded and clearly labelled together in a Word document with the revised version of the manuscript. See Information for Authors for further details.

- The Journal of Physiology funds authors of provisionally accepted papers to use the premium BioRender site to create high resolution schematic figures. Follow this link and enter your details and the manuscript number to create and download figures. Upload these as the figure files for your revised submission. If you choose not to take up this offer, we require figures to be of similar quality and resolution. If you are opting out of this service to authors, state this in the Comments section on the Detailed Information page of the submission form. The link provided should only be used for the purposes of this submission. Authors will be charged for figures created on this premium BioRender account if they are not related to this manuscript submission.

- Please upload separate high-quality figure files via the submission form.

- Please ensure that any tables are editable and in Word format, and wherever possible, embedded in the article file itself.

- Please ensure that the Article File you upload is a Word file.

- Your paper contains Supporting Information of a type that we no longer publish, including supplementary tables and figures. Any information essential to an understanding of the paper must be included as part of the main manuscript and figures. The only Supporting Information that we publish are video and audio, 3D structures, program codes and large data files. Your revised paper will be returned to you if it does not adhere to our Supporting Information Guidelines.

In line with our policy, supplemental tables/figures should be included in the main manuscript wherever they are essential to understanding the paper. If inclusion of this material would interrupt the 'flow' of the paper, they can be included at the bottom as part of an Appendix, instead. Any figures and tables should be numbered as Fig. A1, Fig. A2, Table A1, etc. - and each should be cited in the text.

- Papers must comply with the Statistics Policy: https://jp.msubmit.net/cgi-bin/main.plex?form_type=display_requirements#statistics.

In summary:

- If n {less than or equal to} 30, all data points must be plotted in the figure in a way that reveals their range and distribution. A bar graph with data points overlaid, a box and whisker plot or a violin plot (preferably with data points included) are acceptable formats.

- If n > 30, then the entire raw dataset must be made available either as supporting information, or hosted on a not-for-profit repository, e.g. FigShare, with access details provided in the manuscript.

- 'n' clearly defined (e.g. x cells from y slices in z animals) in the Methods. Authors should be mindful of pseudoreplication.

- All relevant 'n' values must be clearly stated in the main text, figures and tables.

- The most appropriate summary statistic (e.g. mean or median and standard deviation) must be used. Standard Error of the Mean (SEM) alone is not permitted.

- Exact p values must be stated. Authors must not use 'greater than' or 'less than'. Exact p values must be stated to three significant figures even when 'no statistical significance' is claimed.

- All Abstract Figures should be created using BioRender. Authors should use The Journal's premium BioRender account to export high-resolution images. Details on how to use and access the premium account are included as part of this email.

- Authors are required to fully disclose the use of any Generative AI (GAI) tools used in the preparation of their manuscript. This disclosure should also be included in the 'Methods' section (or a suitable location for article types not requiring Methods), and include the name(s), model and version of AI technology used, and exact details on the purpose for, and methods of, its use.

EDITOR COMMENTS

Please also see 'Required Items' above.

Reviewing Editor:

Comments to the Author (Required):
Both specialist reviewers are in agreement on the importance of the findings presented in this work.

They have noted some minor revisions that if implemented would improve the readability and impact of this manuscript.

Senior Editor:

Comments for Authors to ensure the paper complies with the Statistics Policy (Required):
Precise p values must be given.

REFEREE COMMENTS

Referee #1:

This current paper presents a rigorous and mechanistically sophisticated investigation into sex-specific vagal neurocircuit regulation of gastric function by oxytocin (OXT), stress paradigms, and estrous status. Using an integrated experimental framework that combines electrophysiology, in vivo gastric motility recordings, neuronal tracing, immunohistochemistry, and gastric emptying assays, the authors demonstrate that female rats exhibit fundamentally different OXT-dependent modulation of dorsal motor nucleus of the vagus (DMV) neurons compared with previously described male circuitry. Most notably, OXT suppresses inhibitory GABAergic transmission to DMV neurons under basal conditions in females, independent of estrous stage, suggesting constitutive engagement of stress-related CRF signaling pathways in female vagal neurocircuits.

The study is highly relevant to the field of gut-brain interactions and provides important translational insight into why stress-related disorders of gut-brain interaction (DGBI) disproportionately affect women. The experiments are generally well designed, statistically appropriate, and logically organized. The findings regarding tonic CRF1 receptor activity in females, differential recruitment of NANC vagal efferent pathways, and dissociation between central synaptic modulation and peripheral gastric outcomes are novel and physiologically important. Overall, this is an elegant and important contribution to the neurophysiology of gut-brain signaling that substantially advances understanding of sex-specific autonomic regulation and stress responsiveness.

Minor Points

1. Introduction: change "many of which are disproportionately affect women" to "many of which disproportionately affect women."

2. The manuscript alternates between "HE/LE," "high estrogen/low estrogen," and "estrous stage." Consistent terminology throughout the Results and figure legends would improve clarity, particularly for readers outside reproductive neurobiology.

3. Clarify the rationale for grouping estrous stages. The Methods combine diestrus/metestrus as "LE" and proestrus/estrus as "HE." Additional physiological justification or serum estradiol correlation data would strengthen this classification strategy.

4. Clarify whether "N" refers to cells or animals in each figure/table. Although this is described generally in the Methods, the figure legends and tables should explicitly indicate whether sample size represents neurons or rats. This is especially important in electrophysiological datasets where multiple cells may originate from the same animal.

5. The statistical approach for electrophysiology would benefit from a brief clarification.

The manuscript uses both paired t-tests and χ^2 analyses for response proportions, but it is unclear whether corrections for multiple comparisons were considered given the large number of subgroup analyses. A brief justification would help.

6. Improve consistency in nomenclature and formatting of pharmacological agents. Examples include "OXT," "OXY," and "oxytocin" appearing interchangeably. Similarly, "Astressin+OXT" and "Astressin + OXT" are inconsistently formatted.

7. Figure legends could better emphasize the key physiological conclusions. Adding one concise interpretive sentence would help readers rapidly appreciate the biological significance of each experiment. Figures 3 and 4 would particularly benefit from this approach.

8. The Discussion should more explicitly address translational implications for female-predominant DGBI. The mechanistic findings are strong, but the manuscript could better connect the observed tonic CRF signaling and altered vagal recruitment to conditions such as functional dyspepsia, gastroparesis, or IBS in women. This would broaden the clinical impact of the work.

9. Minor typographical and formatting issues should be corrected. Examples include spacing inconsistencies ("17 β-estradiol"), occasional duplicated wording, and several figure/table formatting artifacts. There are also isolated notation inconsistencies for units and symbols (e.g., "mIPSc" vs "mIPSC").

Referee #2:

This remarkably comprehensive study combines whole cell recordings and animal experiments to uncover sex-specific organization of vagal neurocircuitry and OXT modulation of inhibitory input to DMV neurones, while showing that estrous stage and stress determine peripheral effector recruitment. The original findings shed considerable insights on the intersection of sex differences and stress on gastric motility.

1) With studies like this, it is often challenging to present findings while maintaining the appropriate balance between the forest and the trees. This study appropriately addresses multiple interacting factors, including sex differences, low vs high estrus states, astressin and oxytocin, homo and heterotypic stress, inhibitory and excitatory pathways, and integration of these findings with prior work. Perhaps there is an opportunity to focus more on the trees in the Results and the forest in the Discussion.

2) The methods and findings are appropriately detailed. However, several sentences convey multiple concepts, seemed overly long, and are challenging to understand. I would encourage you to avail of the opportunity to make this a more reader-friendly paper. For example, the following sentence "In male rats, CRF1 receptors on vagal neurocircuits are not tonically active under basal (naïve, or unstressed, conditions); following acute stress, however, CRF1 receptors are activated, 'uncovering' the ability of OXT to act presynaptically to inhibit GABAergic NTS-DMV transmission, resulting in DMV 'disinhibition', an increase in vagal efferent output, and an increase in gastric motility and tone" could perhaps be

simplified as follows: "In male rats, CRF1 receptors on vagal circuits are inactive under normal conditions but become activated during acute stress. This allows oxytocin (OXT) to reduce inhibitory signaling between the NTS and DMV, increasing vagal output and thereby increasing gastric motility and tone." Please simplify sentence structure wherever feasible.

3) The introduction mentions prior studies showing that GABA has inhibitory effects on DMV neurones and excitatory cholinergic output from the DMV. Might it be useful to integrate these earlier findings into the Discussion?

4) The relevance of these findings to humans is mentioned only in the Conclusions. Might the Discussion further address the implications of these findings for stress- and possibly pregnancy-related effects on gastric motility in humans? For example, if the finding that "female rats do not respond to stress with any change in GE" also applies to humans, the implications are substantial.

5) Graphical abstract. The uppermost right panel is arguably the most important.

a) I may be mistaken but I believe that this panel attempts to depict the tonic effects of CRF, the effects of astressin and oxytocin. Perhaps it may be simpler to comprehend if this figure is split into 3 figures which show baseline state and effects of perturbations separately.

b) The uppermost panel says "Disinhibition of DMV neuron". Middle panel says "Decreased gastric tone and motility." When I first read that, I could not reconcile "disinhibition" with decreased motility. Perhaps you mean "Disinhibition of DMV-mediated inhibitory activity"?

c) Middle panel does not show cholinergic fibers, which is mentioned in the Introduction. Might that puzzle some readers?

6) Table 2 (p20). The lower panel appears to be cut off.

END OF COMMENTS

This current paper presents a rigorous and mechanistically sophisticated investigation into sex-specific vagal neurocircuit regulation of gastric function by oxytocin (OXT), stress paradigms, and estrous status. Using an integrated experimental framework that combines electrophysiology, in vivo gastric motility recordings, neuronal tracing, immunohistochemistry, and gastric emptying assays, the authors demonstrate that female rats exhibit fundamentally different OXT-dependent modulation of dorsal motor nucleus of the vagus (DMV) neurons compared with previously described male circuitry. Most notably, OXT suppresses inhibitory GABAergic transmission to DMV neurons under basal conditions in females, independent of estrous stage, suggesting constitutive engagement of stress-related CRF signaling pathways in female vagal neurocircuits.

The study is highly relevant to the field of gut-brain interactions and provides important translational insight into why stress-related disorders of gut-brain interaction (DGBI) disproportionately affect women. The experiments are generally well designed, statistically appropriate, and logically organized. The findings regarding tonic CRF1 receptor activity in females, differential recruitment of NANC vagal efferent pathways, and dissociation between central synaptic modulation and peripheral gastric outcomes are novel and physiologically important. Overall, this is an elegant and important contribution to the neurophysiology of gut–brain signaling that substantially advances understanding of sex-specific autonomic regulation and stress responsiveness.

Minor Points

1. Introduction: change “many of which are disproportionately affect women” to “many of which disproportionately affect women.”

2. The manuscript alternates between “HE/LE,” “high estrogen/low estrogen,” and “estrous stage.” Consistent terminology throughout the Results and figure legends would improve clarity, particularly for readers outside reproductive neurobiology.
3. Clarify the rationale for grouping estrous stages. The Methods combine diestrus/metestrus as “LE” and proestrus/estrus as “HE.” Additional physiological justification or serum estradiol correlation data would strengthen this classification strategy.
4. Clarify whether “N” refers to cells or animals in each figure/table. Although this is described generally in the Methods, the figure legends and tables should explicitly indicate whether sample size represents neurons or rats. This is especially important in electrophysiological datasets where multiple cells may originate from the same animal.
5. The statistical approach for electrophysiology would benefit from a brief clarification. The manuscript uses both paired t-tests and χ^2 analyses for response proportions, but it is unclear whether corrections for multiple comparisons were considered given the large number of subgroup analyses. A brief justification would help.
6. Improve consistency in nomenclature and formatting of pharmacological agents. Examples include “OXT,” “OXY,” and “oxytocin” appearing interchangeably. Similarly, “Astressin+OXT” and “Astressin + OXT” are inconsistently formatted.
7. Figure legends could better emphasize the key physiological conclusions. Adding one concise interpretive sentence would help readers rapidly appreciate the biological significance of each experiment. Figures 3 and 4 would particularly benefit from this approach.
8. The Discussion should more explicitly address translational implications for female-predominant DGBI. The mechanistic findings are strong, but the manuscript could better

connect the observed tonic CRF signaling and altered vagal recruitment to conditions such as functional dyspepsia, gastroparesis, or IBS in women. This would broaden the clinical impact of the work.

9. Minor typographical and formatting issues should be corrected. Examples include spacing inconsistencies ("17 β-estradiol"), occasional duplicated wording, and several figure/table formatting artifacts. There are also isolated notation inconsistencies for units and symbols (e.g., "mIPSc" vs "mIPSC").

We would like to thank the editors and reviewers for their encouraging comments regarding our manuscript. Below, we have addressed each comment individually, and the resulting changes are noted in the revised manuscript in red

Referee #1:

1. Introduction: change "many of which are disproportionately affect women" to "many of which disproportionately affect women."
In the Introduction we have changed the sentence to state "many of which disproportionately affect women".

2. The manuscript alternates between "HE/LE," "high estrogen/low estrogen," and "estrous stage." Consistent terminology throughout the Results and figure legends would improve clarity, particularly for readers outside reproductive neurobiology.
We checked for consistency and have amended the manuscript to use HE, LE, OVX, OVX+HE and OVX+LE terminology throughout.

3. Clarify the rationale for grouping estrous stages. The Methods combine diestrus/metestrus as "LE" and proestrus/estrus as "HE." Additional physiological justification or serum estradiol correlation data would strengthen this classification strategy.
We have clarified the rationale for grouping estrous stages in the Methods section, specifically "Due to their relatively lower circulating levels of estrogen levels, diestrus and metestrus were combined as "LE", whereas proestrus and estrus were combined as "HE" due to elevated or peak estrogen level. This classification provides a functional framework for comparing rats with ovariectomized groups receiving estrogen replacement via slow-release capsule containing either a physiologically high or low concentration of 17β-estradiol (180μg/ml HE or 18μg/ml LE, respectively)". We have also included information to note that serum estradiol level was measured to confirm the classification of the 5 groups (HE, LE, OVX, OVX+HE and OVX+LE).

4. Clarify whether "N" refers to cells or animals in each figure/table. Although this is described generally in the Methods, the figure legends and tables should explicitly indicate whether sample size represents neurons or rats. This is especially important in electrophysiological datasets where multiple cells may originate from the same animal.
We have clarified the sample size designation in the revised manuscript. Specifically, 'n' represents the number of neurons/cells recorded, while 'N' represents the number of animals used in each experiment. This information has been added to the Methods section under Statistical Analysis. In addition, all figure legends and tables have been revised to explicitly indicate whether the reported sample size refers to neurons (n) or rats/animals (N), particularly for electrophysiological datasets where multiple neurons may originate from the same animal.

5. The statistical approach for electrophysiology would benefit from a brief clarification. The manuscript uses both paired t-tests and χ^2 analyses for response proportions, but it is unclear whether corrections for multiple comparisons were considered given the large number of subgroup analyses. A brief justification would help.
In electrophysiology experiments, paired t-tests were performed for comparisons within individual neurons to evaluate drug-induced changes relative to baseline, while χ^2 analyses were used to compare the proportions of responsive neurons. Because these analyses were hypothesis-driven and designed to address specific questions rather than to conduct an exploratory screen across all possible comparisons, formal correction for multiple comparisons was not applied. We have clarified this rationale and added a brief justification in the Statistical Analysis section for electrophysiology results in the revised manuscript.

6. Improve consistency in nomenclature and formatting of pharmacological agents. Examples include "OXT," "OXY," and "oxytocin" appearing interchangeably. Similarly, "Astressin+OXT" and "Astressin + OXT" are inconsistently formatted.
The nomenclature and formatting of pharmacological agents have been revised for consistency throughout the manuscript. Specifically, the terms "OXT" is now used consistently, and formatting inconsistencies such as "Astressin+OXT" versus "Astressin + OXT" have been corrected across the text, figures, and figure legends.

7. Figure legends could better emphasize the key physiological conclusions. Adding one concise interpretive sentence would help readers rapidly appreciate the biological significance of each experiment. Figures 3 and 4 would particularly benefit from this approach.
We appreciate this suggestion. We have added a concise sentence at the end of each figure legend to highlight the physiological significance of the findings and to facilitate rapid interpretation of the experimental outcomes. Figures 3 and 4 in particular have been revised to more explicitly emphasize their key biological conclusions."

8. The Discussion should more explicitly address translational implications for female-predominant DGBI. The mechanistic findings are strong, but the manuscript could better connect the observed tonic CRF signaling and altered vagal recruitment to conditions such as functional dyspepsia, gastroparesis, or IBS in women. This would broaden the clinical impact of the work.
We thank the reviewer for highlighting the important translational relevance of this preclinical study. We have amended the Discussion section to address this more explicitly. Specifically, we have included the following:When discussing NTS-DMV neurocircuits and control of gastric functions: "This provides a potential mechanistic explanation as to why circulating ovarian hormone levels alone are poor predictors of DGBI symptom severity in the female population".
When discussion effects of estrogen on OXT-mediated effects: "This provides a potential mechanistic explanation for why circulating ovarian hormone levels alone poorly predict symptom severity associated with DGBI in female populations"
When discussing DGBI symptoms in females: "This may be especially relevant to the clinical observations of DGBI symptoms such as bloating, altered motility, and abdominal pain in female patients which are exacerbated by stress rather than hormonal state alone"
When discussing stress related neuroplasticity: "Not only may this provide a potential mechanistic framework not only for the persistence and reoccurrence of stress to exacerbate upper GI symptoms in female-predominant DGBI, but may also explain the increased dysregulation of gastrointestinal function reported during pregnancy in response to alterations in gonadal hormones as well as stress and anxiety. Clearly, while potentially translationally relevant, the results of the current study require further validation and testing in rodents as well as in in the clinical population".

9. Minor typographical and formatting issues should be corrected. Examples include spacing inconsistencies ("17 β-estradiol"), occasional duplicated wording, and several figure/table formatting artifacts. There are also isolated notation inconsistencies for units and symbols (e.g., "mIPSc" vs "mIPSC").
We thank the reviewer for noticing these typographical and formatting issues. We have carefully reviewed the manuscript and corrected spacing inconsistencies, removed instances of duplicated wording across the text, and corrected unit and symbol inconsistencies, including ensuring uniform use of "mIPSC" throughout the manuscript. All figure and table formatting artifacts have also been addressed in the revised version.

Referee #2:

1) With studies like this, it is often challenging to present findings while maintaining the appropriate balance between the forest and the trees. This study appropriately addresses multiple interacting factors, including sex differences, low vs high estrus states, astressin and oxtytocin, homo and heterotypic stress, inhibitory and excitatory pathways, and integration of these findings with prior work. Perhaps there is an opportunity to focus more on the trees in the Results and the forest in the Discussion.
We thank the reviewer for this suggestion. We have attempted to focus on describing results in the relevant section while providing a 'bigger picture' overview in the Discussion.

2) The methods and findings are appropriately detailed. However, several sentences convey multiple concepts, seemed overly long, and are challenging to understand. I would encourage you to avail of the opportunity to make this a more reader-friendly paper. For example, the following sentence "In male rats, CRF1 receptors on vagal neurocircuits are not tonically active under basal (naïve, or unstressed, conditions); following acute stress, however, CRF1 receptors are activated, 'uncovering' the ability of OXT to act presynaptically to inhibit GABAergic NTS-DMV transmission, resulting in DMV 'disinhibition', an increase in vagal efferent output, and an increase in gastric motility and tone" could perhaps be simplified as follows: "In male rats, CRF1 receptors on vagal circuits are inactive under normal conditions but become activated during acute stress. This allows oxytocin (OXT) to reduce inhibitory signaling between the NTS and DMV, increasing vagal output and thereby increasing gastric motility and tone." Please simplify sentence structure wherever feasible.
We thank the reviewer for this suggestion and have edited the manuscript to simplify sentence structure wherever possible.

3) The introduction mentions prior studies showing that GABA has inhibitory effects on DMV neurons and excitatory cholinergic output from the DMV. Might it be useful to integrate these earlier findings into the Discussion?
We have followed the Reviewer's suggestion to incorporate discussions of the critical role of GABA on vagal efferent motoneuron excitability earlier in the Discussion.

4) The relevance of these findings to humans is mentioned only in the Conclusions. Might the Discussion further address the implications of these findings for stress- and possibly pregnancy-related effects on gastric motility in humans? For example, if the finding that "female rats do not respond to stress with any change in GE" also applies to humans, the implications are substantial.
As in our response to Reviewer 1, we have added additional discussion of the potential translational relevance of these preclinical studies to the clinical population, and have added a brief discussion on pregnancy-related alterations in gastric function. Specifically, we note that: "Not only may this provide a potential mechanistic framework not only for the persistence and reoccurrence of stress to exacerbate upper GI symptoms in female-predominant DGBI, but may also explain the increased dysregulation of gastrointestinal function reported during pregnancy in response to alterations in gonadal hormones as well as stress and anxiety. Clearly, while potentially translationally relevant, the results of the current study require further validation and testing in rodents as well as in the clinical population".

5) Graphical abstract. The uppermost right panel is arguably the most important.

a) I may be mistaken but I believe that this panel attempts to depict the tonic effects of CRF, the

effects of astressin and oxytocin. Perhaps it may be simpler to comprehend if this figure is split into 3 figures which show baseline state and effects of perturbations separately.

b) The uppermost panel says "Disinhibition of DMV neuron". Middle panel says "Decreased gastric tone and motility." When I first read that, I could not reconcile "disinhibition" with decreased motility. Perhaps you mean "Disinhibition of DMV-mediated inhibitory activity"?

c) Middle panel does not show cholinergic fibers, which is mentioned in the Introduction. Might that puzzle some readers?
As per the Editor's request, the graphical abstract has been re-drawn in BioRender, and we have taken this opportunity to incorporate the Reviewer's suggestions.

6) Table 2 (p20). The lower panel appears to be cut off.
Table 2 appears complete in the revised manuscript.

Dear Dr Browning,

Re: JP-RP-2026-291603R1 "**Estrogen modulates stress-induced vagally-dependent gastric responses to oxytocin**" by Ruchi Bhagat, R Alberto Travagli, and Kirsteen N Browning

Thank you for submitting your manuscript to The Journal of Physiology. It has been assessed by a Reviewing Editor and by 2 expert referees and we are pleased to tell you that it is acceptable for publication following satisfactory revision.

Please share this decision with your co-authors as soon as possible.

The referee reports are copied at the end of this email.

Please address all the points raised and incorporate all requested revisions or explain in your Response to Referees why a change has not been made. We hope you will find the comments helpful and that you will be able to return your revised manuscript within 4 weeks. If you require longer than this, please contact journal staff: jp@physoc.org.

Your revised manuscript should be submitted online using the link in your Author Tasks Link Not Available. This link is accessible via your account as Corresponding Author; it is not available to your co-authors. If this presents a problem, please contact journal staff (jp@physoc.org). Image files from the previous version are retained on the system. Please ensure you replace or remove any files that are being revised.

If you do not wish to submit a revised version of your manuscript, you must inform our journal staff (jp@physoc.org) or reply to this email to request withdrawal. Please note that a manuscript must be formally withdrawn from the peer review process at one journal before it may be submitted to another journal.

TRANSPARENT PEER REVIEW POLICY: To improve the transparency of its peer review process The Journal of Physiology publishes online, as supporting information, the peer review history of all articles accepted for publication. Readers will have access to decision letters, including Editors' comments and referee reports, for each version of the manuscript, as well as any author responses to peer review comments. Referees can decide whether or not they wish to be named on the peer review history document.

ABSTRACT FIGURES: Authors are expected to use The Journal's premium BioRender account to create/redraw their Abstract Figures. Information on how to access this account is here: https://physoc.onlinelibrary.wiley.com/journal/14697793/biorender-access.

This will enable Authors to create and download high-resolution figures. If authors have used the free BioRender service, they can use the instructions provided in the link above to download a high-resolution version suitable for publication.

The link provided should only be used for the purposes of this submission. Authors will be charged for figures created on this account if they are not related to this manuscript submission.

LANGUAGE EDITING AND SUPPORT FOR PUBLICATION: If you would like help with English language editing, or other article preparation support, Wiley Editing Services offers expert help, including English Language Editing, as well as translation, manuscript formatting, and figure formatting at www.wileyauthors.com/eeo/preparation. You can also find resources for Preparing Your Article for general guidance about writing and preparing your manuscript at www.wileyauthors.com/eeo/prepresources.

RIGOUR AND REPRODUCIBILITY: We recommend authors consult The Journal's Rigour and Reproducibility requirements to ensure that their manuscript is suitable for publication: https://physoc.onlinelibrary.wiley.com/pb-assets/hub-assets/physoc/documents/TJP-Rigour-and-Reproducibility-Requirements.pdf

REVISION CHECKLIST:

Check that your Methods section conforms to journal policy: https://jp.msubmit.net/cgi-bin/main.plex?form_type=display_requirements#methods

Check that data presented conforms to the statistics policy: https://jp.msubmit.net/cgi-bin/main.plex?form_type=display_requirements#statistics

Upload a full Response to Referees file. To create your 'Response to Referees' copy all the reports, including any comments from the Senior and Reviewing Editors, into a Microsoft Word, or similar, file and respond to each point, using font or background colour to distinguish comments and responses and upload as the required file type.

Please upload two versions of your manuscript text: one with all relevant changes highlighted and one clean version with no changes tracked. The manuscript file should include all tables and figure legends, but each figure/graph should be uploaded as separate, high-resolution files. The journal is now integrated with Wiley's Image Checking service. For further details,

see: https://www.wiley.com/en-us/network/publishing/research-publishing/trending-stories/upholding-image-integrity-wileys-image-screening-service

You may also upload:

- 'Potential Cover Art' for consideration as the issue's cover image
- Appropriate Supporting Information (Video, audio or data set: see https://jp.msubmit.net/cgi-bin/main.plex?form_type=display_requirements#supp).

We look forward to receiving your revised submission.

If you have any queries, please reply to this email and we will be pleased to advise.

Yours sincerely,

Kim Barrett
Senior Editor
The Journal of Physiology

REQUIRED ITEMS

- The Journal of Physiology funds authors of provisionally accepted papers to use the premium BioRender site to create high resolution schematic figures. Follow this link and enter your details and the manuscript number to create and download figures. Upload these as the figure files for your revised submission. If you choose not to take up this offer, we require figures to be of similar quality and resolution. If you are opting out of this service to authors, state this in the Comments section on the Detailed Information page of the submission form. The link provided should only be used for the purposes of this submission. Authors will be charged for figures created on this premium BioRender account if they are not related to this manuscript submission.

- Your paper contains a Supporting Information section, which can be deleted, as you have incorporated this material as an Appendix, which is consistent with our requirements (it will be included in the manuscript). The only Supporting Information that we publish are video and audio, 3D structures, program codes and large data files. Please see our Supporting Information Guidelines.

EDITOR COMMENTS

Please also see 'Required Items' above.

Reviewing Editor:

Methods Details:
Specific details of euthanasia should be provided (use of anaesthesia and following cardiac puncture noted) but euthanasia should be explicitly stated.

Comments to the Author (Required):
Both reviewers have considered this manuscript and view it favourably in terms of the impact of its findings. However, reviewer 2 has identified improvements in language use and flow, which could be simplified and thereby would make it more accessible to readers.

REFEREE COMMENTS

Referee #2:

The edits have improved the manuscript, but it remains a dense read. This is partly due to the complexity of the topic and the sentence structure, where there is still considerable room for improvement. Addressing these issues, however, would require substantial revisions. I identified only one sentence as an example, while noting that similar opportunities likely exist throughout the manuscript. I've highlighted a few examples below. There are several others throughout the manuscript.

Abstract

1) A simplified and perhaps more accurate version of "The brain-gut axis is central to gastrointestinal (GI) regulation and the severity of disorders of gut-brain interaction (DGBI) are more prevalent in females" is "The brain-gut axis is central to gastrointestinal (GI) regulation, and disorders of gut-brain interaction (DGBI) are more prevalent and often more severe in females." (The "severity" is not "more prevalent").

2) A simpler and more accurate version of "The aim of the present study was to test the hypothesis that the brain-gut neurocircuitry reveals sex-specific organization in response to stress and ovarian hormone levels" is "The aim of the present study was to test the hypothesis that brain-gut neurocircuitry in female rats is influenced by stress and ovarian hormone levels." (To claim sex-specific organization, you would need to study male and female rats).

3) A simplified version of "Using the 13C breath-test in Sprague-Dawley female rats revealed that gastric emptying was slower during periods of high levels of estrogen, but stress had no further effect to delay gastric emptying" is "In female Sprague-Dawley rats, gastric emptying was slower when estrogen levels were high, but stress did not slow it further."

4) Rather than "Whole-cell recordings revealed that, in contrast to male rats, the "anti-stress" peptide, oxytocin (OXT), suppressed GABAergic transmission to dorsal motor nucleus of the vagus (DMV) neurons under non-stressed conditions independently of estrus stage", I might say "Whole-cell recordings showed that, unlike what has been reported in male rats, oxytocin (OXT) reduced GABAergic signaling to dorsal motor nucleus of the vagus (DMV) neurons in female rats under non-stressed conditions, regardless of estrous cycle stage." (Male rats were not studied in this paper).

5) This (very dense) sentence "Application of astressin, an antagonist of the pro-stress corticotropin-releasing factor (CRF), revealed that portions of the presynaptic effects of OXT were determined by the tonically active CRF1 receptors in female vagal neurocircuits" can be simplified to read "Application of Astressin, a corticotropin-releasing factor (CRF) receptor antagonist, showed that some of oxytocin's presynaptic effects depend on ongoing CRF1 receptor activity in female vagal neurocircuits."

6) The title of the graphical abstract refers to "sex-specific differences," but only female rats were studied in this paper. This wording therefore seems to overstate the findings. In my view, the graphical abstract should convey the main message on its own, but at present it is not sufficiently clear. The top panel includes oxytocin, CRF, and Astressin, which appear to lead to downstream effects; however, the specific relationships among these factors and how they support the main conclusion need to be clarified. I would redo the abstract/

I would encourage you to make these edits because (a) the findings are important and (b) a more reader-friendly version would likely reach a wider audience. Using AI programs, such edits can be made effectively and efficiently.

END OF COMMENTS

We thank the editors and reviewers for their additional comments, particularly regarding the density of the sentence structure. We have addressed each of their suggestions (below) and made changes in the manuscript (red).

Methods Details:

Specific details of euthanasia should be provided (use of anaesthesia and following cardiac puncture noted) but euthanasia should be explicitly stated.

We have noted that "Female rats were euthanized following anesthesia with isoflurane (5% in air) truncal blood was collected via cardiac puncture and processed for plasma collection for later analysis. Following a bilateral pneumothorax" and "rats were euthanized via administration of a bilateral pneumothorax and transcardial perfusion".

Referee #2:

Abstract

1) A simplified and perhaps more accurate version of "The brain-gut axis is central to gastrointestinal (GI) regulation and the severity of disorders of gut-brain interaction (DGBI) are more prevalent in females" is "The brain-gut axis is central to gastrointestinal (GI) regulation, and disorders of gut-brain interaction (DGBI) are more prevalent and often more severe in females." (The "severity" is not "more prevalent").

We have amended the abstract as suggested.

2) A simpler and more accurate version of "The aim of the present study was to test the hypothesis that the brain-gut neurocircuitry reveals sex-specific organization in response to stress and ovarian hormone levels" is "The aim of the present study was to test the hypothesis that brain-gut neurocircuitry in female rats is influenced by stress and ovarian hormone levels." (To claim sex-specific organization, you would need to study male and female rats).

In principle, we agree with the reviewer but previous studies from our group studied male rats and the results of the present manuscript are explicitly compared to those prior studies and the results of the present study do suggest differences in neurocircuit organization. Regardless, we agree the suggested phrasing is more easily understood so we have amended the abstract as suggested.

3) A simplified version of "Using the 13C breath-test in Sprague-Dawley female rats revealed that gastric emptying was slower during periods of high levels of estrogen, but stress had no

further effect to delay gastric emptying" is "In female Sprague-Dawley rats, gastric emptying was slower when estrogen levels were high, but stress did not slow it further."
We have amended the abstract to note: Gastric emptying rats in Sprague–Dawley female rats were slower when estrogen levels were high, but stress had no further effect

4) Rather than "Whole-cell recordings revealed that, in contrast to male rats, the "anti-stress" peptide, oxytocin (OXT), suppressed GABAergic transmission to dorsal motor nucleus of the vagus (DMV) neurons under non-stressed conditions independently of estrus stage", I might say "Whole-cell recordings showed that, unlike what has been reported in male rats, oxytocin (OXT) reduced GABAergic signaling to dorsal motor nucleus of the vagus (DMV) neurons in female rats under non-stressed conditions, regardless of estrous cycle stage." (Male rats were not studied in this paper).
As above, the results of this study were explicitly compared to prior studies in males, but we have amended the manuscript to note that Whole-cell recordings revealed that, unlike previous reports in male rats, the "anti-stress" peptide, oxytocin (OXT), suppressed GABAergic transmission to dorsal motor nucleus of the vagus (DMV) neurons under non-stressed conditions, regardless of estrogen level

5) This (very dense) sentence "Application of astressin, an antagonist of the pro-stress corticotropin-releasing factor (CRF), revealed that portions of the presynaptic effects of OXT were determined by the tonically active CRF1 receptors in female vagal neurocircuits" can be simplified to read "Application of Astressin, a corticotropin-releasing factor (CRF) receptor antagonist, showed that some of oxytocin's presynaptic effects depend on ongoing CRF1 receptor activity in female vagal neurocircuits."
We have amended the abstract to note that Application of Astressin, an antagonist of the "pro-stress" neuropeptide, corticotropin-releasing factor (CRF), showed that some presynaptic effects of OXT were dependent upon ongoing CRF1 receptor activity in female vagal neurocircuits.

6) The title of the graphical abstract refers to "sex-specific differences," but only female rats were studied in this paper. This wording therefore seems to overstate the findings. In my view, the graphical abstract should convey the main message on its own, but at present it is not sufficiently clear. The top panel includes oxytocin, CRF, and Astressin, which appear to lead to downstream effects; however, the specific relationships among these factors and how they support the main conclusion need to be clarified. I would redo the abstract/
We have changed the title of the graphical abstract to "Effects of Estrogen and Stress on Brain-Gut Neurocircuits".

2nd Revision - Editorial Decision 12-Aug-2026

Dear Dr Browning,

Re: JP-RP-2026-291603R2 "**Estrogen modulates stress-induced vagally-dependent gastric responses to oxytocin**" by Ruchi Bhagat, R Alberto Travagli, and Kirsteen N Browning

We are pleased to tell you that your paper has been accepted for publication in The Journal of Physiology.

The last Word (or similar) version of the manuscript provided will be used by the Production Editor to prepare your proof. When this is ready you will receive an email containing a link to Wiley's Online Proofing System. The proof should be thoroughly checked and corrected as promptly as possible.

Authors should note that it is too late at this point to offer corrections prior to proofreading. Major corrections at proof stage, such as changes to figures, will be referred to the Editors for approval before they can be incorporated. Only minor changes, such as to style and consistency, should be made at proof stage. Changes that need to be made after proof stage will usually require a formal correction notice.

All queries at proof stage should be sent to: TJP@wiley.com.

Following copyediting and typesetting the accepted version will be published online.

Yours sincerely,

Kim Barrett
Senior Editor
The Journal of Physiology

IMPORTANT POINTS TO NOTE FOLLOWING ACCEPTANCE OF YOUR PAPER:

• IMPORTANT NOTICE ABOUT OPEN ACCESS: To assist authors whose funding agencies mandate immediate public access to published research findings, The Journal of Physiology allows authors to pay an Open Access (OA) fee to have their papers made freely available immediately on publication.

The Corresponding Author will receive an email from Wiley with details on how to register or log in to Wiley Authors where you will be able to place an order.

• You can check if your funder or institution has a Wiley Open Access Account here:
https://authors.wiley.com/author-resources/Journal-Authors/open-access/author-compliance-tool.html

MEMBER DISCOUNT: Corresponding authors who are Members of the Physiological Society are eligible for a 20% discount on the cost of the Article Processing Charge (APC). To claim this discount, the Corresponding Author will need to log in to the Physiological Society Membership portal and retrieve the discount code (https://www.physoc.org/journals-and-media/journals/journal-discount-for-members/). This code should then be quoted in the billing section of Wiley Authors when selecting the open access option.

• TRANSPARENT PEER REVIEW POLICY: To improve the transparency of its peer review process, The Journal of Physiology publishes online as supporting information the peer review history of all articles accepted for publication. Readers will have access to decision letters, including Editors' comments and referee reports, for each version of the manuscript as well as any author responses to peer review comments. Referees can decide whether or not they wish to be named on the peer review history document.

• You can help your research get the attention it deserves! Check out Wiley's free Promotion Guide for best-practice recommendations for promoting your work at: www.wileyauthors.com/eeo/guide. You can learn more about Wiley Editing Services which offers professional video, design, and writing services to create shareable video abstracts, infographics, conference posters, lay summaries, and research news stories for your research at: www.wileyauthors.com/eeo/promotion.

• If you would like to receive our 'Research Roundup', a monthly newsletter highlighting the cutting-edge research published in The Physiological Society's family of journals (The Journal of Physiology, Experimental Physiology, Physiological Reports, The Journal of Nutritional Physiology and The Journal of Precision Medicine: Health and Disease), please click this link, fill in your name and email address and select 'Research Roundup':
https://www.physoc.org/journals-and-media/membernews

EDITOR COMMENTS

Reviewing Editor:

Comments to the Author (Required):
All points raised by the reviewer have been satisfactorily addressed.
